# Tropism of Extracellular Vesicles and Cell-Derived Nanovesicles to Normal and Cancer Cells: New Perspectives in Tumor-Targeted Nucleic Acid Delivery

**DOI:** 10.3390/pharmaceutics13111911

**Published:** 2021-11-11

**Authors:** Anastasiya Oshchepkova, Oleg Markov, Evgeniy Evtushenko, Alexander Chernonosov, Elena Kiseleva, Ksenia Morozova, Vera Matveeva, Lyudmila Artemyeva, Valentin Vlassov, Marina Zenkova

**Affiliations:** 1 Institute of Chemical Biology and Fundamental Medicine SB RAS, 630090 Novosibirsk, Russia; oshchepkova.2018@gmail.com (A.O.); markov_ov@niboch.nsc.ru (O.M.); alexander.chernonosov@niboch.nsc.ru (A.C.); vam@niboch.nsc.ru (V.M.); mila_a@ngs.ru (L.A.); valentin.vlassov@niboch.nsc.ru (V.V.); 2 Faculty of Chemistry, Lomonosov Moscow State University, 119234 Moscow, Russia; evtushenko@enzyme.chem.msu.ru; 3 Institute of Cytology and Genetics SB RAS, 630090 Novosibirsk, Russia; elka@bionet.nsc.ru (E.K.); morozova.kn@gmail.com (K.M.)

**Keywords:** extracellular vesicles, vesicle mimetic, nucleic acid delivery, cytochalasin B, mesenchymal stem cells, dendritic cells, apoptosis, tumor cells

## Abstract

The main advantage of extracellular vesicles (EVs) as a drug carrier system is their low immunogenicity and internalization by mammalian cells. EVs are often considered a cell-specific delivery system, but the production of preparative amounts of EVs for therapeutic applications is challenging due to their laborious isolation and purification procedures. Alternatively, mimetic vesicles prepared from the cellular plasma membrane can be used in the same way as natural EVs. For example, a cytoskeleton-destabilizing agent, such as cytochalasin B, allows the preparation of membrane vesicles by a series of centrifugations. Here, we prepared cytochalasin-B-inducible nanovesicles (CINVs) of various cellular origins and studied their tropism in different mammalian cells. We observed that CINVs derived from human endometrial mesenchymal stem cells exhibited an enhanced affinity to epithelial cancer cells compared to myeloid, lymphoid or neuroblastoma cancer cells. The dendritic cell-derived CINVs were taken up by all studied cell lines with a similar efficiency that differed from the behavior of DC-derived EVs. The ability of cancer cells to internalize CINVs was mainly determined by the properties of recipient cells, and the cellular origin of CINVs was less important. In addition, receptor-mediated interactions were shown to be necessary for the efficient uptake of CINVs. We found that CINVs, derived from late apoptotic/necrotic cells (aCINVs) are internalized by in myelogenous (K562) 10-fold more efficiently than CINVs, and interact much less efficiently with melanocytic (B16) or epithelial (KB-3-1) cancer cells. Finally, we found that CINVs caused a temporal and reversible drop of the rate of cell division, which restored to the level of control cells with a 24 h delay.

## 1. Introduction

Extracellular vesicles (EVs) are known to participate in cancer development [1]; in the spread of bacterial, viral, prion, and other infections [2]; and in neurodegenerative and mental disorders [3], cardiovascular diseases [4], and others. They have found application in diagnostics and in therapeutic design. The ability of EVs to carry various cargos provides a possibility to design EV-based drug delivery systems [5,6]. However, there are some difficulties in their practical therapeutic use. First, there are different ways of EV internalization by recipient cells [7,8,9], and current knowledge is insufficient to predict the cellular localization of an EV cargo [10] and the effectiveness of therapy. Second, the application of EVs for drug delivery requires modification of the vesicles with targeting molecules [5,9,11]. Such engineering may have multiple negative effects, such as vesicle aggregation, damage to the EV structure, and increased immunogenicity [5,12]. Finally, processes of natural EV isolation and purification are quite laborious. Therefore, attempts have been made to produce various artificial mimetics of EVs [12,13]. The preparation of EV-mimetics is often aimed at the production of exosome-like vesicles ~100 nm in size. One of the simplest ways to produce EV mimetics is to treat cells with cytochalasins.

Cytochalasins are a group of structurally similar compounds with pronounced anti-cancer effects. They disrupt microfilaments by blocking actin polymerization [14], which leads to the disorganization of various actin-containing filament structures in cells [15]. Cytochalasin B (Cyt B) is best known as a prospective chemotherapy drug [14]. In addition to acting on microfilaments, Cyt B inhibits glucose transporters [16]. Cell sensitivity to Cyt B can vary significantly [17], which is especially noticeable in the first hours after treatment [18]. A different cellular response could be expected even when similar types of tumors were treated [19]. In cancer cells, Cyt B induces apoptosis, probably via both pathways [19,20,21,22]. However, apoptosis progression induced by Cyt B happens slowly, as the transfer of phosphatidylserine to the outer layer of the plasma membrane does not occur significantly, even 12–24 h after exposure [20,21]. Moreover, a 3 h treatment of cells with Cyt B still permits the identification of some tumor-associated proteins on the cell surface [23], despite observed changes of cell morphology [20].

Initially, Cyt B-inducible vesicles were shown to be useful as cell mimetics [24,25]. Cell treatment with Cyt B leads to the formation of tubular protrusions on the plasma membrane, from which micro- and nanometer-sized membrane vesicles can be separated by shaking [24]. Here, we name these mimetic vesicles cytochalasin-B-inducible nanovesicles (CINVs) according to our previous report [26]. CINVs were used to monitor complex biochemical signaling cascades that arise, for example, after exposure to olfactory [27,28,29,30,31] or taste [32,33,34] receptors or to test the interaction of drugs with P-glycoprotein, a transmembrane protein associated with the emergence of the multiple drug resistance phenotype of tumor cells [35]. CINVs preserve the membrane and part of the cytoplasm of their parental cells [36], which allows them to respond to external stimuli [37]. The preservation of the integrity of CINV membrane gave rise to the idea of using them for drug delivery [38,39,40,41]. The encapsulation of therapeutics into CINVs increased their biocompatibility and retention time in tumors and the blood [40,42,43,44]. To increase the CINV affinity to tumor cells, a nucleolin-specific aptamer AS1411 or folic acid was used [40,43].

In the case of nucleic acid delivery, the effectiveness of therapy largely depends on the appropriate drug localization in the cells. Recent research has shown that CINVs can fuse with recipient cells [36], which may provide cytoplasmic drug localization that is convenient for nucleic acid-based therapeutics. To load a DNA-oligonucleotide into the CINVs, we previously used a freezing/thawing method [26]. Alternatively, reversible membrane permeability with digitonin followed by the restoration of membrane integrity by adding Ca^2+^ [40,42,43,44] can also be used for nucleic acid loading into nanovesicles.

The choice of the cellular source for CINV preparations is important in the case of nanovesicle use as delivery vectors. The intrinsic content of CINVs may mediate effects similar to those of their parent cells, for example, enhance angiogenesis, as observed for CINVs derived from SH-SY5Y neuroblastoma cells [45] or mesenchymal stem cells [36]. Moreover, given the pro-apoptotic effect of Cyt B, CINVs should also be well-characterized for potential cytotoxicity, as animal experiments demonstrated their strong non-specific accumulation in the lung and liver [40,43,46], even if the vesicles were conjugated with tumor-targeting molecules [40,43].

In this study, we investigated the interactions of nano-sized CINVs and EVs loaded with fluorescein labelled oligodeoxynucleotides with cancer cells of different origins: the selectivity of CINV internalizations by the cells, the role of protein–protein interactions in CINVs uptake, and the cytotoxicity of CINVs.

## 2. Materials and Methods

### 2.1. Reagents

Dulbecco’s modified Eagle’s medium (DMEM), Roswell Park Memorial Institute-1640 (RPMI-1640), Iscove’s modified Dulbecco’s medium (IMDM), 2-mercaptoethanol, sodium pyruvate, Histopaque-1083 medium, formaldehyde, and HEPES were purchased from Sigma (St. Louis, MO, USA). Fetal bovine serum (FBS) was purchased from GE Healthcare Life Science HyClone Laboratories (Logan, UT, USA). Fetal bovine serum mesenchymal stem cell-qualified (FBS MSC-qualified) and Versen solution were purchased from Biolot (St. Petersburg, Russia). TrypLE™ Express Enzyme, Opti-Minimal Essential Medium (Opti-MEM), and Glutamax were purchased from Gibco (Thermo Fisher Scientific, Waltham, MA, USA). Trypsin-EDTA solution, antibiotic/antimycotic solution (10,000 IU/mL Penicilin-10 mg/mL Streptomycin-25 µg/mL Amphotericin B), and phosphate buffered saline (PBS) were purchased from MP Biomedicals, LCC (Illkirch, France). For nanoparticle tracking analysis, PBS was purchased from Gibco (Cat. 70011036, Thermo Fisher Scientific, Waltham, MA, USA).

Lipofectamine 2000 and aldehyde/sulfate latex beads (A37304) were purchased from Invitrogen (Carlsbad, CA, USA). Granulocyte macrophage-colony stimulating factor (GM-CSF) and interleukin-4 (IL-4) were purchased from Sino Biological Inc. (Beijing, China). Cytochalasin B was purchased from AppliChem GmbH (Darmstadt, Germany). Mung Bean nuclease (Cat. M0250L) and NEB buffer were purchased from New England Biolabs (Ipswich, MA, USA). The WST-1 cell proliferation assay kit was purchased from Takara Bio Inc. (Kusatsu, Japan). The Qubit protein assay Kit and ProLong™ Glass Antifade Mountant with NucBlue™ were purchased from Thermo Fisher Scientific (Eugene, OR, USA). Proteinase K PCR grade (#EO0491) was purchased from Thermo Fisher Scientific (Vilnius, Lithuania).

The following antibodies were purchased from Sony Biotechnology (San Jose, CA, USA) and used to stain CINVs derived from human endometrial mesenchymal stem cells (MSCs): CD9 (Cat. 2160520, fluorescein isothiocyanate (FITC) mouse IgG1 κ), CD63 (Cat. 2365040, allophycocyanin (APC) mouse IgG1 κ), CD81 (Cat. 2347530, phycoerythrin (PE) mouse IgG1 κ), CD14 (Cat. 2228015, FITC mouse IgG1 κ), CD19 (Cat. 2111025, FITC mouse IgG1 κ), CD34 (Cat. 2317525, PE mouse IgG1 κ), CD45 (Cat. 2120025, FITC mouse IgG1 κ), CD90 (Cat. 2240565, APC mouse IgG1 κ), and CD105 (Cat. 2216025 PE mouse IgG1 κ). The CD73 was purchased from Biolegend (San Diego, CA, USA) (Cat. 334015, FITC mouse IgG1 κ). The isotype controls used in these experiments were purchased from Sony Biotechnology (San Jose, CA, USA): Cat. 2600550, FITC mouse IgG1 κ; Cat. 2600570, PE mouse IgG1 κ; Cat. 2600610, APC mouse IgG1 κ. 

The following antibodies were purchased from Sony Biotechnology (San Jose, CA, USA) and used to stain dendritic cell (DC)-derived EVs or CINVs and tsDCs: CD63 (Cat. 1319520, PE Rat IgG2a κ), CD81 (Cat. 1124550, APC Hamster IgG), CD80 (Cat. 1123570, APC Hamster IgG), and CD86 (Cat. 1125155, Brilliant Violet 421 (BV421) Rat IgG2a κ). The MHC II was purchased from Abcam (Cambridge, UK) (ab15638, FITC Rat IgG2b). Two isotype controls used in these experiments were purchased from Sony Biotechnology (San Jose, CA, USA): Cat. 2602540, PE Rat IgG2a κ and Cat. 2604560, APC Hamster IgG.

Palloidin-iFluor 532 Reagent (ab176755) was purchased from Abcam (Cambridge, UK). Annexin V Binding Buffer (Cat. 2711005), Annexin V-FITC (Cat. 3804530), and 7-amino-actinomycin D viability staining solution (7-AAD) (Cat. 2702020) were purchased from Sony Biotechnology (San Jose, CA, USA).

### 2.2. Materials

In addition, 25 cm^2^ and 150 cm^2^ cell culture flasks, 0.22 µm syringe filter, and 24- and 96-well plates were purchased from TPP (Trasadingen, Switzerland). Twelve-well plates were purchased from Orange scientific (Braine-l’Alleud, Belgium). Amicon Ultra 0.5 mL Ultracel 3K columns were purchased from Millipore Corp. (Burlington, MA, USA). Sixteen-well E-plates were purchased from ACEA Biosciences, Inc. (San Diego, CA, USA).

### 2.3. Cells

HEK 293, KB-3-1, A549, IMR-32, MCF-7, HepG2, J774, HL-60, L929, and K562 were purchased from the Institute of Cytology RAS (St. Petersburg, Russia). B16 was provided by the National Medical Research Center of Oncology named after N.N. Blokhin (Moscow, Russia). Raw 264.7 was provided by Prof. D.V. Kuprash (Engelhardt Institute of Molecular Biology, RAS, Moscow, Russia). KELLY, THP-1, Jurkat, and tsDCs were purchased from the European Collection of Authenticated Cell Cultures (ECACC) (Salisbury, UK). Raji and Daudi were provided by Dr. A.V. Stepanov (M.M. Shemyakin and Yu. A. Ovchinnikov Institute of Bioorganic Chemistry, RAS, Moscow, Russia). RLS was provided by Prof. N.A. Popova (Institute of Cytology and Genetics Siberian Branch of RAS, Novosibirsk, Russia).

All cell lines were cultivated in medium supplemented with 1% antibiotic/antimycotic mix. The cultivation of cells at 37 °C and 5% CO_2_ (for tsDCs: 33 °C, 5–7.5% CO_2_) is indicated further in the text as cultivation at standard conditions.

HEK 293, KB-3-1, A549, IMR-32, MCF-7, HepG2, J774, B16, and L929 cells were grown in DMEM supplemented with 10% FBS. Kelly, THP-1, and Jurkat cells were grown in RPMI-1640 supplemented with 10% FBS and 2 mM Glutamax. K562, Raji, and Daudi cells were grown in RPMI-1640 supplemented with 10% FBS. HL-60 cells were cultivated in IMDM supplemented with 20% FBS. The tsDC cells were grown in IMDM supplemented with 5% FBS, 50 µM 2-mercaptoethanol, and 2 mM Glutamax. RLS cells were grown in IMDM supplemented with 10% FBS. Raw 264.7 cells were cultivated in DMEM (4.5 g/L glucose concentration) supplemented with 10% FBS and 1 mM sodium pyruvate. The growth media presented above are indicated in the text as the respective cell culture media.

Human endometrial mesenchymal stem cells (MSCs) were isolated from healthy donors and cultivated in IMDM supplemented with 10% FBS MSC-qualified, 2 mM Glutamax and were characterized as previously described [26]. The donors provided informed consent in accordance with the rules of the local ethics committee. Primary immature dendritic cells (DCs) were obtained from the bone marrow of C57Bl/6 mice by centrifugation in Histopaque-1083 medium, as previously reported [47]. Briefly, 10^6^ cells/mL were cultivated in IMDM supplemented with 10% FBS, 50 ng/mL GM-CSF, and 50 ng/mL IL-4.

### 2.4. Isolation of Natural Extracellular Vesicles (EVs)

DC-derived EVs were isolated from the conditioned medium of mice bone marrow-derived DCs or tsDCs by differential centrifugation, as previously reported [26]. Briefly, when tsDCs reached ~90% confluence or after the 6-day cultivation of bone marrow-derived DCs, the medium was replaced with FBS-free medium supplemented with 0.5% human albumin. After 2 days of cultivation, conditioned medium was collected and subjected to several centrifugations at 300× *g* (10 min, 4 °C), 2000× *g* (15 min, 4 °C), 12,000× *g* (30 min, 4 °C), and 100,000× *g* (70 min, 4 °C) (Beckman Coulter, Avanti J-301, JA 30.50 Ti rotor, Brea, CA, USA). The EV-pellet was washed with 10 mL TBS (20 mM Tris-HCl, pH 7.5, and 150 mM NaCl) and centrifuged overnight at 100,000× *g*, at 4 °C. The resultant pellet was dissolved in TBS and stored at 4 °C for no longer than 7 days. All procedures were performed under sterile conditions.

The yield of EVs was estimated as total protein concentration (see Section 2.6). One mg of the total EV protein can be isolated from 7.8 ± 1.9 × 10^8^ immature bone marrow-derived DCs (*n* = 4) or from 17.7 ± 6.5 × 10^8^ tsDCs (*n* = 5).

### 2.5. Isolation of Cytochalasin-B-Inducible Nanovesicles (CINVs) from Live or Apoptotic Cells

The CINVs prepared from late apoptotic/necrotic cells are indicated by the prefix “a”, whereas those from live cells have no prefix. All procedures were performed under sterile conditions. Cytochalasin B (Cyt B) stock solution was prepared in dimethyl sulfoxide (DMSO), aliquoted, and stored at −20 °C until use.

The procedure of CINV preparation was carried out as previously described [26]. Adhesive cells, which achieved 100% confluence, were collected by Versen solution, except for Raw 264.7 and L929 cells, which were collected by cell spatula. Approximately 50–100 × 10^6^ cells were placed into a 25 cm^2^ cell culture flask with a vented cap in 5 mL of fresh FBS-free medium that contained Cyt B at concentration 10 µg/mL. Cells were incubated under standard conditions for 30 min. The flask was then vigorously vortexed for 30 s, the CINVs were collected into 1.5 mL tubes and subjected to several consecutive centrifugations at 100× *g* (10 min, 4 °C), at 600× *g* (20 min, 4 °C, twice), and 15,000× *g* (30 min, 4 °C). The pellet, obtained after 15,000× *g*, was washed with 1 mL TBS and subjected to centrifugation at 15,000× *g* (30 min, 4 °C). The CINV pellet was resuspended in TBS and stored at −80 °C until use.

To produce aCINVs, cell death was induced before nanovesicle preparation. Cells were incubated in respective cell culture medium supplemented with 1% antibiotic/antimycotic and H_2_O_2_ at concentrations in a range of 0.25–50 mM for 24 h; B16 and KB-3-1 cells were used when 100% confluence was achieved, and K562 cells were seeded at a density 0.5 × 10^6^ cells/mL.

The yield of CINVs was estimated as total protein concentration (see Section 2.6). One milligram of CINVs (total protein) was obtained from 1.3 ± 0.7 × 10^8^ MSCs (*n* = 8), 1.5 ± 1.1 × 10^8^ tsDCs (*n* = 3), 1.7 ± 0.4 × 10^8^ HEK 293 (*n* = 7), 2.1 ± 0.6 × 10^8^ IMR-32 (*n* = 5), 0.9 ± 0.1 × 10^8^ KB-3-1 (*n* = 4), 2.1 ± 0.8 × 10^8^ L929 (*n* = 4), 2.3 ± 0.9 × 10^8^ Raw 264.7 (*n* = 4), 1.0 ± 0.3 × 10^8^ K562 (*n* = 4), and 1.6 ± 0.3 × 10^8^ B16 (*n* = 3).

One milligram of aCINVs (total protein) was obtained from 2.1 × 10^8^ B16 (0.25 mM H_2_O_2_, *n* = 2), 2.6 × 10^8^ B16 (1 mM H_2_O_2_, *n* = 2), 0.5 × 10^8^ KB-3-1 (1 mM H_2_O_2_, *n* = 2), 0.9 × 10^8^ KB-3-1 (10 mM H_2_O_2_, *n* = 2), 3.0 × 10^8^ K562 (1 mM H_2_O_2_, *n* = 2), and 4.2 ± 1.7 × 10^8^ K562 (50 mM H_2_O_2_, *n* = 4).

### 2.6. Qubit Protein Assay, Dynamic Light Scattering (DLS), and Nanoparticle Tracking Analysis (NTA)

The measurement of total protein concentration was performed using Qubit protein assay Kit and Qubit 2.0 Fluorimeter according to manufacture recommendations, as previously reported [26]. Samples were previously lysed in 0.5% sodium dodecyl sulfate (SDS) for 15 min at room temperature. Fluorescence was measured at 485/510–580 nm.

The size and number of EVs and CINVs were measured by dynamic light scattering (DLS) and nanoparticle tracking analysis (NTA). Nanovesicle samples were prepared in 6 × filtered TBS/PBS for the measurements. Buffers were filtered through a 0.22 µm syringe filter into 50 mL pre-sterile centrifuge tubes under sterile conditions. DLS was performed using Zetasizer Nano ZS (Malvern Instruments, Malvern, UK) in backscattering mode (173°). NTA was performed using a Nanosight LM10 HS-BF instrument (Nanosight Ltd., Salisbury, UK) equipped with a 405 nm laser (65 mW) and high sensitivity EMCCD camera (Andor Luca, Belfast, UK). Measurements were made according to the recommendations of ASTM E2834-12 (2018) [48] using NTA 2.3 software (Nanosight Ltd., Salisbury, UK) with optimized camera and video processing setups [49,50]. Briefly, samples of EVs/CINVs were diluted in particle-free PBS to achieve optimal concentration of about 1.5 × 10^8^ particles/mL. Each sample was measured seven times, in two technical replicates (N = 14, at least 5000 total tracks; video length was 60 sec). Sets of tracks from all recorded videos were merged to produce the joint particle size distribution, mean particle size, and total particle concentration, corrected by dilution factor.

To determine the level of EV or CINV sample contamination with non-vesicular protein or particles, the nanovesicle isolation procedure was performed in the absence of cells. The amount of non-vesicular protein in EVs was calculated for samples obtained from 30–200 mL (*n* = 3) of culture medium subjected to all steps of EV isolation. The number of non-vesicular particles in the EVs determined by NTA was analyzed in samples obtained from 30 mL of cell culture medium and was about 10^9^ particles.

The number of non-vesicular particles in the CINVs was analyzed for the sample obtained from a 150 cm^2^ cell culture flask. It was ~10^8^ particles/mL. Similarly, the concentration of particles in the buffer used for CINV preparation was ~10^8^ particles/mL.

### 2.7. Staining with Surface Protein Markers

To study the surface protein markers of EVs or CINVs, nanovesicles were immobilized on 4 µm aldehyde/sulfate latex beads as reported [26] and analyzed using the NovoCyte™ flow cytometer (ACEA Biosciences, Inc., San Diego, CA, USA).

For antibody staining, the tsDCs were detached by Versen solution, washed with PBS (0.01 M phosphate, 0.0027 M KCl and 0.137 M NaCl, pH ~ 7.4 at 25 °C), washed with a blocking solution (PBS + 10% EV-depleted FBS + 0.5% BSA), and incubated in the blocking solution for 15 min at room temperature. Antibodies were used according to the manufacturer’s recommendations. Cell staining was performed in the blocking solution for 30 min at room temperature.

The EV-depleted FBS was prepared by overnight centrifugation at 100,000× *g* (4 °C). FITC was excited at 488 nm and detected at 530 ± 30 nm; PE was excited at 488 nm and detected at 572 ± 28 nm; APC was excited at 640 nm and detected at 675 ± 30 nm; BV421 was excited at 405 nm and detected at 445 ± 45 nm (Pacific Blue channel) (NovoCyte™). At least two independent experiments were performed for each staining.

### 2.8. Loading of EVs and CINVs with FAM-Labeled Oligodeoxyribonucleotide (FAM-ON)

Oligodeoxyribonucleotide (5′-AGT-CTC-GAC-TTG-CTA-CC-3′), bearing fluorescein at the 5′-end (FAM-ON), was synthesized by standard phosphatidamide solid phase protocol, deprotected, and isolated by HPLC. The homogeneity of FAM-ON was confirmed to be ≥95%.

The loading of nanovesicles with FAM-ON was performed by three cycles of sample freezing in liquid nitrogen, followed by thawing as described previously [26]. Briefly, 50 µg EVs or 15/50 µg CINVs/aCINVs and 1 nmol FAM-ON were dissolved in TBS or FBS-free medium used for each particular cell culture: imDEX or tsDEX–in 20–40 µL TBS, tsDC CINVs–in 20 µL TBS, other CINVs/aCINVs–in 20 µL medium. Samples were frozen in liquid nitrogen, stored at −80 °C for 10 min, thawed in a water bath (25 °C) and vigorously shaken for 10 min at 700 rpm (25 °C). The procedure was trice repeated. After the third freezing, the samples could be left overnight at −80 °C, then thawed, shaken again (10 min at 700 rpm, 25 °C), and added to the cells.

### 2.9. FAM-ON Delivery into Cells

All experiments were performed in 24-well plates in antibiotic/antimycotic free medium. Adhesive cells were collected by trypsin-ethylenediaminetetraacetic acid (EDTA) solution (0.25% trypsin and 1 mM EDTA) or TrypLE™ express enzyme; tsDCs were collected by Versen solution. Adhesive and semi-adhesive cells were pre-seeded at a density providing 10^5^ cells/well at the start of the experiment and left to adhere overnight. Suspension cells were seeded on the day of the experiment at a density of 10^5^ cells/well.

Experiments with EVs were performed in 250 µL Opti-MEM supplemented with 1 mM sodium pyruvate and 5 mM HEPES (pH 7.3) for 8 h under standard conditions. Experiments with CINVs/aCINVs were performed in 250 µL of the respective cell culture medium (supplemented with EV-depleted FBS) for 4 h under standard conditions. These conditions were chosen according to our previous research [26].

To detect the level of FAM-ON self-penetration (CTRO), the oligonucleotide (1 nmol in 250 µL) was added to cells in the absence of nanovesicles. Lipofectamine 2000 delivery of FAM-ON was used as a positive control (LF) and was performed according to the manufacturer’s recommendations. Briefly, 1 µL of Lipofectamine 2000 was dissolved in 24 µL Opti-MEM and incubated for 5 min at room temperature. Next, 0.25 nmol FAM-ON dissolved in 25 µL Opti-MEM was added, mixed and incubated at room temperature for 20 min. Finally, the complexes were added to 10^5^ cells and incubated in FBS-free medium for 4 h under standard conditions (final volume = 250 µL).

After incubation, cells were collected using trypsin-EDTA solution or TrypLE™ express enzyme, fixed with 3.7% formaldehyde and analyzed by NovoCyte™ flow cytometer. The following gating strategy was used: SSC-A vs. FSC-A to identify cells by granularity/size and FSC-H vs. FSC-A to exclude cell doublets. Each experiment was repeated independently 2–6 times, and the total number of measurements (n) ranged from 3 to 9.

### 2.10. Annexin V and 7-AAD Viability Assay

To test different conditions of apoptosis induction, cells were incubated in a 12-well plate in the respective cell culture medium supplemented with H_2_O_2_ (from 0.25 mM to 50 mM) for 24 h. B16 and KB-3-1 cells were used when 100% confluence was achieved. K562 cells were seeded at a density of 0.5 × 10^6^ cells/mL on the day of the experiment. After those cells were collected, they were washed with Annexin V Binding Buffer and stained with 7-AAD and Annexin V-FITC according to the manufacturer’s recommendations. After staining, cells were placed on ice and measured as soon as possible. The 7-AAD was excited at 488 nm and detected at 675 ± 30 nm by NovoCyte™ flow cytometer. Annexin V-FITC was excited at 488 nm and detected at 530 ± 30 nm (NovoCyte™). At least two independent experiments were performed for each condition.

### 2.11. Proteinase K and Trypsin-EDTA Assays

All experiments were performed in antibiotic/antimycotic free medium.

Proteinase K assay: B16 cells were pre-seeded in a 24-well plate and left to adhere overnight, as described in Section 2.9. The 10^5^ cells/well were washed with fresh DMEM and incubated in 250 µL FBS-free DMEM supplemented with 100 µg/mL proteinase K, 1 mM sodium pyruvate, and 5 mM HEPES for 30 min under standard conditions. After that, the medium was carefully removed, cells were washed with fresh DMEM, and FAM-ON delivery was performed immediately, as described in Section 2.9. Three independent experiments were performed.

Trypsin-EDTA assay: B16 cells were seeded in 25 cm^2^ cell culture flask to achieve 100% confluence. The growth medium was removed; cells were washed with PBS and incubated with 1 mL trypsin-EDTA (0.25% trypsin and 1 mM EDTA) for 4 min under standard conditions. Next, 4 mL of fresh DMEM supplemented with 10% EV-depleted FBS was added. Cells were sedimented at 1000 rpm, resuspended in fresh DMEM supplemented with 10% EV-free FBS, and seeded in 24-well plates at a density of 10^5^ cells/well. The FAM-ON delivery experiments (see Section 2.9) were performed immediately after trypsinization. Three independent experiments were performed.

### 2.12. Confocal Microscopy

HEK293 cells were seeded on glass coverslips in 24-well plates and incubated with MSC CINVs loaded with FAM-ON, as described in Section 2.9. After incubation, coverslips with cells were washed with PBS, cells were fixed with 3.7% formaldehyde for 15 min at 37 °C, washed with PBS, and stained with Palloidin-iFluor 532 Reagent according to the manufacturer’s protocol. Coverslips with cells were placed on a drop of ProLong™ Glass Antifade Mountant with NucBlue™ and mounted samples were allowed to cure on a flat, dry surface for 18–24 h at room temperature in the dark. Confocal imaging was performed with a LSM710 inverted confocal microscope (Zeiss, Oberkochen Germany), using αPlan-Apochromat 100×/1.46 Oil DIC M27 objective. Analysis of the intracellular accumulation of FAM-ON and Z-stack was conducted using ZEN software (Zeiss, Oberkochen Germany). Confocal analysis was performed in three channels (blue, green, red). Fluorescence in the blue channel corresponds to NucBlue™ (nuclei staining), in the green channel corresponds to fluorescence of FAM-ON, and in the red channel corresponds to Phalloidin-iFluor 532 (cytoskeleton staining).

### 2.13. Electron Microscopy

Transmission electron microscopy (TEM) was performed at the Center for Microscopy of Biological Subjects (Institute of Cytology and Genetics SB RAS, Novosibirsk, Russia) using a JEM1400 microscope (80 kV, JEOL, Tokyo, Japan). Negative contrast staining with uranyl acetate, EV and CINV size measurement, and aggregate counting were performed as previously described [26]. Briefly, vesicle sizes were measured using iTEM software (Olympus Soft Imaging Solutions, Münster, Germany). The operator was blinded to the assignment of samples to experimental groups. For each sample, the measurements were performed on 40–50 randomly selected grid squares from two different EM grids. Aggregate counting was performed for 20 randomly selected grid squares for each sample. The number of measured EVs or CINVs in each sample ranged from 150 to 721.

### 2.14. Mass-Spectrometry Analysis (MS)

Mass spectrometry analysis (MS) was carried out at the Center for Mass Spectrometric Analysis (Institute of Chemical Biology and Fundamental Medicine SB RAS, Novosibirsk, Russia). Gradient separation chromatography was performed on an Agilent 1200 HPLC system (Agilent Technologies Inc., Santa Clara, CA, USA) using a Zorbax Eclipse XBD-C18 column (4.6 × 150 mm, internal diameter 5 μm) with an Eclipse XBD-C18 (4.6 × 12.5 mm, inner diameter 5 μm). MS detection was performed on an Agilent 6410 QQQ mass spectrometer (Agilent Technologies Inc., Santa Clara, CA, USA) equipped with an electrospray ionization (ESI) source. The detection was carried out using positive electrospray ionization in the multiple reaction monitoring mode at a spray voltage of 4000 V, a turbo spray temperature of 300 °C, and using nitrogen as a nebulizer gas at 40 psi and a flow rate of 9.2 L/min.

A calibration curve was built for Cyt B concentrations from 1 to 100 ng/mL. Cyt B was extracted from the solution containing HEK 293 membrane-derived nanovesicles (MDNVs) [26] (75 µg) with 300 μL of 0.1% formic acid in methyl alcohol.

For extraction, the 75 µg HEK CINVs in 50 μL TBS were heated to 95 °C and incubated for 15 min with constant mixing at 900 rpm. For extraction, 300 μL of 0.1% formic acid in methyl alcohol was added to this solution. The samples were heated to 60 °C and incubated for 30 min with constant mixing at 900 rpm. Samples were centrifuged using the Amicon Ultra column 0.5 mL Ultracel 3K (Amicon^®^ Ultra-0.5 Centrifugal Filter Concentrator with Ultracel^®^ 3 Regenerated Cellulose Membrane, NMWL: 3,000, volume: 0.5 mL, not sterile) at 13,000 rpm for 20 min. The mixture was then evaporated to dryness and dissolved in 30 μL 0.1% formic acid in methyl alcohol. For analysis, 10 μL of this solution was used.

### 2.15. WST-1 Assay

The colorimetric WST-1 assay was performed according to the manufacturer’s recommendation: cells at a density of 7500 cells/well were seeded in 96-well plates to adhere overnight. Cell incubation with CINVs or Cyt B was performed in 150 µL DMEM supplemented with 10% EV-depleted FBS. Four or thirty micrograms CINVs or 1, 10, 100 ng Cyt B dissolved in 5 µL TBS was added to cells and cells were incubated for 4 h, 24 h, or 48 h. To control cells (CTR), 5 µL TBS was added. WST-1 solution was incubated with the cells for different times: A549 for 1 h and L929 for 2 h. Absorbance was measured at 450 nm and 620 nm was used as a reference wavelength in a Multiskan RC reader (Thermo Labsystems (Thermo Fisher Scientific)). Each experiment was repeated independently 2–3 times.

### 2.16. xCELLigence RTCA

Experiments were carried out using xCELLigence RTCA (ACEA Biosciences, Inc., San Diego, CA, USA): 7500 cells per well were seeded in 16-well E-plate to adhere overnight before the experiment. Cell incubation with CINVs was performed in 150 µL DMEM supplemented with 10% EV-depleted FBS. Thirty micrograms of CINVs dissolved in 5 µL TBS was added to the cells. To control cells (CTR), 5 µL TBS was added. Blank was measured in fresh medium without cells after plate storage at room temperature for 30 min. Cell index was measured every 30 min. Slope was measured starting 2 h after the addition of CINVs to a time indicated in a figure legend. Each experiment was repeated independently 3−4 times.

### 2.17. Mung Bean Nuclease Assay

Fifteen micrograms of HEK CINVs or MSC CINVs and 1 nmol FAM-ON were dissolved in 20 µL TBS and subjected to freezing/thawing, as described in Section 2.8. The nanovesicle solution was then diluted to 200 µL by TBS, and samples were centrifuged at 15,000× *g* for 30 min at 4 °C. The pellet was suspended in 50 µL 1 × NEB buffer (30 mM NaCl, 50 mM sodium acetate, 1 mM ZnSO_4_ (pH 5 at 25 °C)) and treated with 10 U of mung bean nuclease at 30 °C for 1 h. The reaction was stopped by the addition of 5 µL 200 mM EDTA-10 mM ATP. The CINVs were again precipitated by centrifugation at 15,000× *g* for 30 min, resuspended in 50 µL TBS, and absorbed on 4 µm aldehyde/sulfate latex beads (4 µL). The samples incubated in the absence of mung bean nuclease were used as a positive control. Experiments with HEK CINVs and MSC CINVs were performed independently.

### 2.18. Statistical Analysis

Data are presented as the mean and standard deviation (SD), except for NTA data on mean particle size. The latter is represented as mean ± 95% confidence interval (CI_95_), calculated using Student’s t-distribution. Pairwise sample comparison was performed by non-parametric Mann–Whitney U-test.

## 3. Results

### 3.1. General Characterization of Natural EVs and CINVs

Two types of dendritic cells (DCs) were used in this work to produce EVs: mouse immature bone marrow-derived DCs and conditionally immortalized tsDCs [51]. EVs were isolated from the conditioned culture medium of non-stimulated DCs.

The EVs derived from immature bone marrow DCs (imDEX) and tsDCs (tsDEX) were characterized by the expression of some surface protein markers. We observed that imDEX and tsDEX had similar phenotypes (Table 1 and Figure S1A). However, because DC-derived EVs should reflect the immunological status of their parent cells [52,53], we hypothesized that CD80 and MHC II proteins were damaged on the surface of tsDEX during EV isolation by ultracentrifugation. Indeed, the phenotype of tsDCs was more consistent with mature cells (Figure S1B) based on the high levels of CD80 and CD86 expression [54]. Moreover, the yield of tsDEX, measured as total protein concentration, was ~2.3-fold less than that of imDEX (see Section 2.4, Materials and Methods), which was in good agreement with the earlier report showing that mature DCs produce 2- to 3-fold fewer EVs than immature ones [55]. Thus, the imDEX and tsDEX cannot be considered as nanovesicles of a similar type according to phenotype investigation.

**Figure 1:**
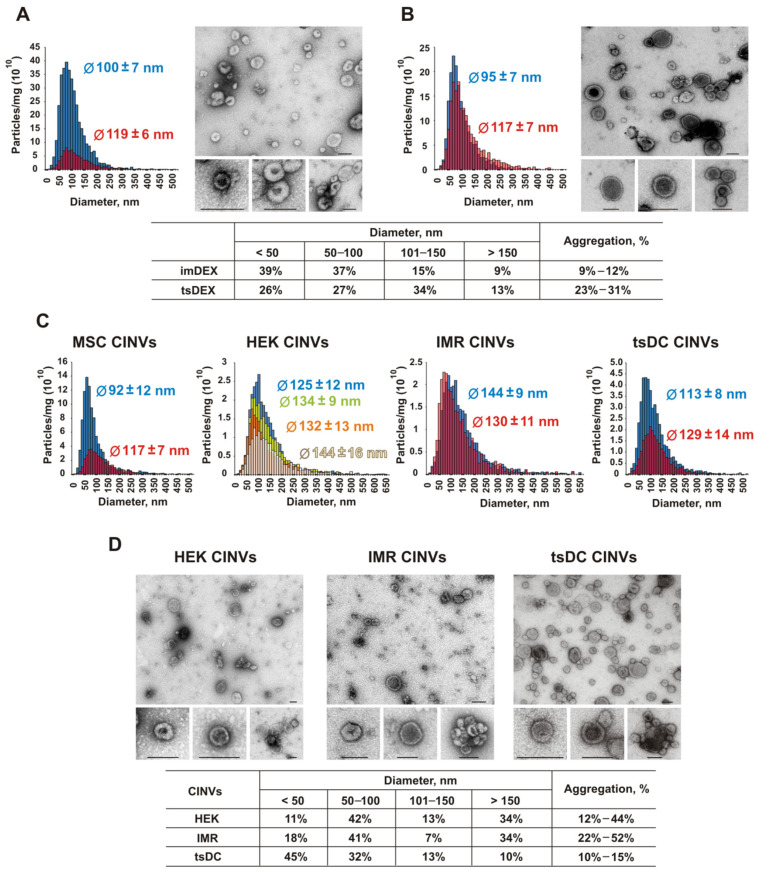
Analysis of size distribution and morphology of EVs and CINVs using NTA and TEM. (**A**)–imDEX, (**B**)–tsDEX, (**C**,**D**)–data, obtained for CINVs, derived from various cell types. Histograms show the size distribution of EV or CINV samples measured by NTA: each color corresponds to data obtained for independently isolated samples. The number of particles was calculated by NTA in 1 mg (total protein) of EVs or CINVs diluted in 1 mL of buffer. The vesicle diameter [Ø] obtained by NTA is presented as the mean ± 95% confidence interval (CI_95_). TEM data are presented as microphotographs with scale bar = 100 nm and tables showing the distribution of the nanovesicles between four groups with diameters [Ø] < 50 nm, 50–100 nm, 101–150 nm, and >150 nm.

The size and aggregation state of EVs were analyzed using nanoparticle tracking analysis (NTA) and transmission electron microscopy (TEM), both number-weighted techniques, as well as dynamic light scattering (DLS). For both tested EV types (imDEX and tsDEX), mean NTA particle sizes were within the 95–120 nm range (Table 1 and Figure 1) typical for EVs, and also showed similar content of particles ≤150 nm on TEM of approximately 90%. These data might be complemented with DLS, which is naturally intensity-weighted and characterizes the upper part of the size distribution along with particle aggregates. Whereas Z-average sizes of imDEX and tsDEX were similar (~200 nm), PdI for the latter EVs was higher. This finding correlates well with the estimation of EVs aggregation state from TEM data, showing low (<15%) aggregation levels for imDEX and moderate (~30%) aggregation levels for tsDEX.

Quantification of vesicles was constructed based on protein concentration measurements and NTA particle concentration data. The yield of EVs was estimated as total protein concentration, which might be represented as a sum of vesicular proteins and contamination with co-isolated non-vesicular proteins (mainly albumin) (1):

Total protein = vesicular proteins + non-vesicular proteins (1)

The amount of non-vesicular protein linearly depended on the volume of cell culture medium and was measured to be 0.36 ± 0.07 µg per 1 mL of medium (for details see Section 2.6, Materials and Methods). The contribution of non-vesicular protein might be considered unchanged when the same volume of conditioned cell medium was used to isolate EVs. Thus, a lower secretory activity of cells causes an increase in the portion of non-vesicular protein in EV samples. In particular, 26 ± 6% of the total protein in imDEX (*n* = 4) was non-vesicular contamination, but in tsDEX, where the yield of nanovesicles was 2.3-fold lower, it reached 54 ± 25% (*n* = 5).

With these considerations kept in mind, one milligram (total protein) of EVs contained 0.8–3.7 × 10^12^ particles in the case of imDEX and 1.8 × 10^12^ particles in the case of tsDEX (Table 1).

Although the amount of non-vesicular protein in EV samples was high, the amount of non-vesicular particles (isolated from non-conditioned medium) was low. The level of imDEX contamination with non-vesicular particles was 1–3% (*n* = 2), whereas in tsDEX it was 1–6% (*n* = 2). This was likely due to the NTA measurement settings used, which were optimized to detect EVs with sizes above 30 nm. Most likely, detected co-isolated proteins were present in a form of small aggregates.

Nine cell types were used in this study to produce CINVs: human endometrial mesenchymal stem cells (MSCs), tsDCs, HEK 293, IMR-32, K562, Raw 264.7, KB-3-1, B16, and L929 cells (Table 1). Similar to EVs, these nanovesicles were characterized by several surface protein markers. We observed that some surface proteins of tsDC CINVs almost disappeared compared to donor cells, whereas CD86 and CD63 were partially saved (Table 1 and Figure S1C). At the same time, the CINVs derived from MSCs retained the phenotype of parent cells [26], except for the CD105 protein, which was not detected on these nanovesicles (Table 1 and Figure S2).

The size, aggregation, and amount of CINVs were also estimated by DLS, NTA, and TEM. According to TEM and NTA, the sizes of CINVs did not differ significantly from the size of natural EVs (Table 1 and Figure 1), whereas the DLS Z-average size was overestimated and indicated a high level of sample heterogeneity (PdI) (Table 1). TEM data show that all studied CINVs have a high level of aggregation (up to 50%), except for tsDC CINVs, which was 10–15% (Figure 1).

The characterization of CINVs samples showed that two samples of MSC CINVs or tsDC CINVs prepared independently differed in particle amounts by a factor of 2.5 (Table 1 and Figure 1). The data obtained for HEK CINVs or IMR CINVs by DLS, NTA, and TEM (Table 1) as well as DLS data obtained for other CINVs correlate well, indicating good reproducibility of the CINV preparation procedure. On average, 1 mg of CINVs contained 0.2–1.0 × 10^12^ particles depending on the source of the nanovesicles (Table 1).

The yield of CINVs was measured as total protein concentration. As the CINVs were produced from cells, the samples mainly contained vesicular proteins, whereas the level of non-vesicular particles in CINV samples was negligible (Section 2.6, Materials and Methods). In this case:

Total protein = vesicular proteins(2)

Thus, our data show that EVs and CINVs have similar number-weighted mean sizes, which generally do not exceed 150 nm. In contrast to EVs, the method of CINV preparation allows nanovesicles with negligible amounts of non-vesicular proteins to be obtained. However, the CINVs are less stable in buffer systems than natural EVs and have a more pronounced tendency to aggregate. The yield of nanovesicles measured either as total protein concentration or as number of particles can vary significantly between the samples of EVs or CINVs, but the factors affecting the yield of nanovesicles remain beyond the scope of this study and need to be investigated.

### 3.2. Characterization of FAM-ON Loaded CINVs

To study the interaction of natural and artificial nanovesicles with recipient cells in vitro and to avoid any disturbances of their surface determinants that could be caused by their labeling with fluorescent dyes, EVs and CINVs were loaded with a fluorescein-labeled single-stranded DNA-oligonucleotide (FAM-ON) by the freezing/thawing method, as previously reported [26]. Using these oligonucleotide-loaded nanovesicles provided us with possibilities (i) to monitor the interaction of nanovesicles with cells by flow cytometry, (ii) to estimate the effectiveness of the nanovesicle uptake by recipient cells, and (iii) to follow the intracellular localization of the cargo (FAM-ON) by confocal microscopy. Self-penetration of free FAM-ON into the cells presented in the samples of nanovesicles was controlled in each experiment (CTRO) because no additional isolation steps of FAM-ON loaded nanovesicles were performed after freezing/thawing procedure and before their adding to cells.

As noted above, the loading of FAM-ON into CINVs was performed by the freezing/thawing method [26]. The internal localization of FAM-ON in CINVs was confirmed by sample treatment with mung bean nuclease followed by flow cytometry assay. To detect the FAM-ON loaded CINVs by flow cytometry, nanovesicles were immobilized on 4 µm aldehyde/sulfate latex beads. Three samples of MSC CINVs and three samples of HEK CINVs, loaded with FAM-ON, were treated with nuclease; no significant reduction of FAM-signal was observed compared to control (non-treated) CINVs (Table S1). The ability of CINVs to deliver FAM-ON into recipient cells was confirmed by confocal microscopy (Figure S3). We also did not observe any significant self-penetration of free FAM-ON (CTRO) into the cells (Figure S4).

### 3.3. Interaction of DC-Derived EVs or CINVs with Various Mammalian Cells

DCs play a crucial role in the induction of adaptive immunity. Naïve immature DCs are activated by tumor-associated antigens, causing their maturation [56]. DCs secrete EVs, bearing classic co-stimulating (CD80 and CD86) and MHC I, MHC II molecules [52,53]. Although DC-derived antigen-bearing EVs can theoretically directly stimulate T-lymphocytes, it is more likely that their main targets are other DCs that have not previously encountered an antigen [57,58,59]. In addition, in vitro experiments demonstrated the ability of cancer cells to internalize EVs secreted by mature DCs and acquire some of their surface proteins [60].

The EVs, derived from immature DCs, are characterized by low immunogenicity and higher yield compared to mature DCs, which makes them attractive for designing a tumor-targeted drug delivery system. However, whether EVs derived from immature DCs can be internalized by cancer cells is not known, as is whether this process is selective and depends on the properties of EVs. Another important question is whether the DC-derived CINVs can be considered an alternative to DC-derived EVs for cancer treatment.

In this study, we focused on the investigation of CINVs derived from immortalized DCs, as the yield of CINVs from tsDCs was much higher compare to bone marrow DCs due to availability of cells. To understand whether tsDCs could be used similar to bone marrow-derived DCs, the interaction of imDEX and tsDEX with different cell types was compared. We used four types of murine cells as recipients of DC-derived EVs or CINVs: non-epithelial skin cancer B16 mouse melanoma cells, RLS lymphosarcoma cells histologically corresponding to diffuse large B-cell lymphoma, J774 macrophage-like cells, and L929 mouse fibroblasts.

**Figure 2:**
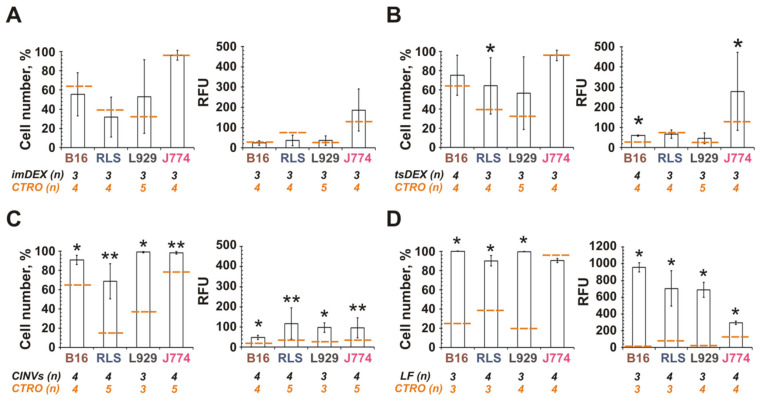
FAM-ON delivery into murine cells mediated by DC-derived EVs or CINVs. Fifty micrograms of FAM-ON loaded imDEX (**A**), tsDEX (**B**) or tsDC CINVs (**C**) were incubated with B16, RLS, L929, or J774 cells. (**D**)–FAM-ON delivery mediated by Lipofectamine 2000 (LF). Cell number is the percentage of FAM-positive cells in a population. RFU is relative fluorescence units detected in a cell population. The level of FAM-ON self-penetration into the cells (CTRO) is shown by an orange dashed line. The number of measurements is indicated as *n*. Experimental data are presented by white columns as the mean ± SD. Statistical analysis was performed using the Mann–Whitney U test and was carried out between the experimental group (*n* = 3–4) and CTRO (*n* = 3–5); * *p* ≤ 0.05, ** *p* ≤ 0.01.

The data obtained showed that FAM-ON-loaded imDEX or tsDEX were unable to efficiently deliver FAM-ON to any of the studied cell lines (Figure 2A,B). In the case of tsDEX, some intracellular accumulation of FAM-ON was observed in J774 and B16 cells, but it was not the case for imDEX. In contrast, tsDC CINVs delivered FAM-ON into all studied cells with comparable efficiency (Figure 2C): in this case, 50–100% of cells in the population were FAM-positive with the mean fluorescence intensity of 50–120 RFU. Thus, tsDC CINVs exhibited unique behavior that was only partially similar to tsDEX. Apparently, the tropism of CINVs and EVs, derived from the same cell type, to various cells may not match completely.

### 3.4. Tropism of MSC-Derived CINVs (MSC CINVs) to Various Mammalian Cells

Because tsDC CINVs exhibited low, if any, selectivity with respect to cell type (Figure 2C), the CINVs derived from another source were analyzed to finally assess their cellular tropism, which provides their targeting properties. The MSC-derived CINVs were chosen as the most interesting mimetic vesicle type for anti-cancer therapy. Non-malignant and cancer cells were incubated with 15 or 50 µg MSC CINVs loaded with FAM-ON followed by the analysis of intracellular FAM-ON accumulation by flow cytometry.

**Figure 3:**
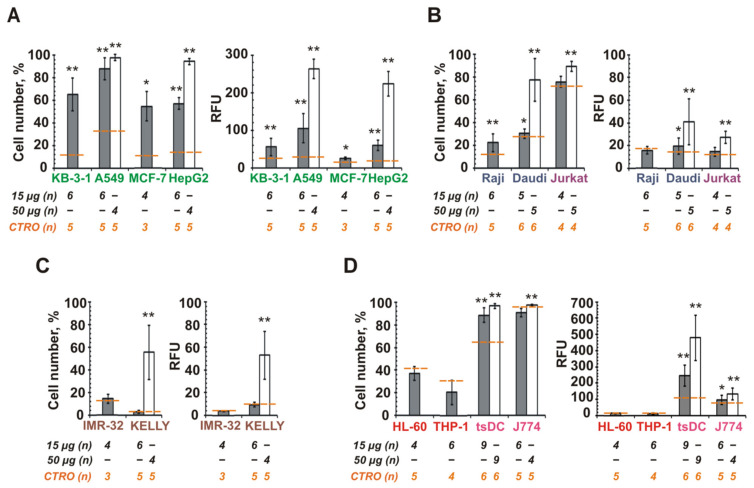
FAM-ON accumulation in mammalian cells mediated by MSC CINVs. Fifteen micrograms (grey bars) or 50 µg (white bars) FAM-ON loaded MSC CINVs were incubated with: (**A**)–Epithelial cancer cells; (**B**)–Lymphoid cancer cells; (**C**)–Human neuroblastoma cells; (**D**)–Human myeloid cancer cells, mouse tsDCs, and macrophage-like J774 cells. Cell number is the percentage of FAM-positive cells in a population. RFU is relative fluorescence units. The level of FAM-ON self-penetration into the cells (CTRO) is shown by an orange dashed line. The number of measurements is indicated as *n*. “-“–not determined. Experimental data are presented as the mean ± SD. Statistical analysis was performed using the Mann–Whitney U test and was carried out between the experimental group (*n* = 4–9) and CTRO (*n* = 3–6); * *p* ≤ 0.05, ** *p* ≤ 0.01.

Epithelial cancer cells effectively internalized the MSC CINVs (Figure 3A). For these cells, the mean level of FAM-ON accumulation increased as follows (15 µg MSC CINVs): MCF-7 (25 RFU) < KB-3-1, HepG2 (55–60 RFU) < A549 (105 RFU). Burkitt’s lymphoma (Raji and Daudi) and T cell leukemia cells (Jurkat) interacted with MSC CINVs much less efficiently than epithelial cancer cells (Figure 3A,B). Only when a high dose of MSC CINVs (50 µg) was used were detectable levels of FAM-ON accumulation in cancer lymphoid cells observed. However, it was 6- to 10-fold less efficient than CINV-mediated FAM-ON uptake by epithelial cells (Figure 3A,B, RFU, 50 µg).

In the case of neuroblastoma cells, the detectable level of FAM-signal was observed in KELLY cells incubated with 50 µg FAM-ON loaded MSC CINVs (Figure 3C). The MSC CINVs were effectively internalized by tsDCs and by macrophage-like J774 cells (Figure 3D). Of note, the levels of CINV uptake by J774 were similar for nanovesicles derived from both MSCs and tsDCs (Figure 2C and Figure 3D). Finally, human THP-1 or HL-60 leukemia cells were unable to internalize MSC CINVs (Figure 3D).

Thus, the internalization of MSC CINVs by studied cells was more selective than in the case of tsDC CINVs. We observed that MSC CINVs demonstrated a higher affinity for epithelial cancer cells. Whether these differences in the behavior of MSC CINVs and tsDC CINVs were determined by their different cellular origins, the preservation of the integrity of their surface proteins, or the properties of recipient cells remained unclear.

### 3.5. The Role of CINV Origin in Interaction with Cancer Cells

**Figure 4:**
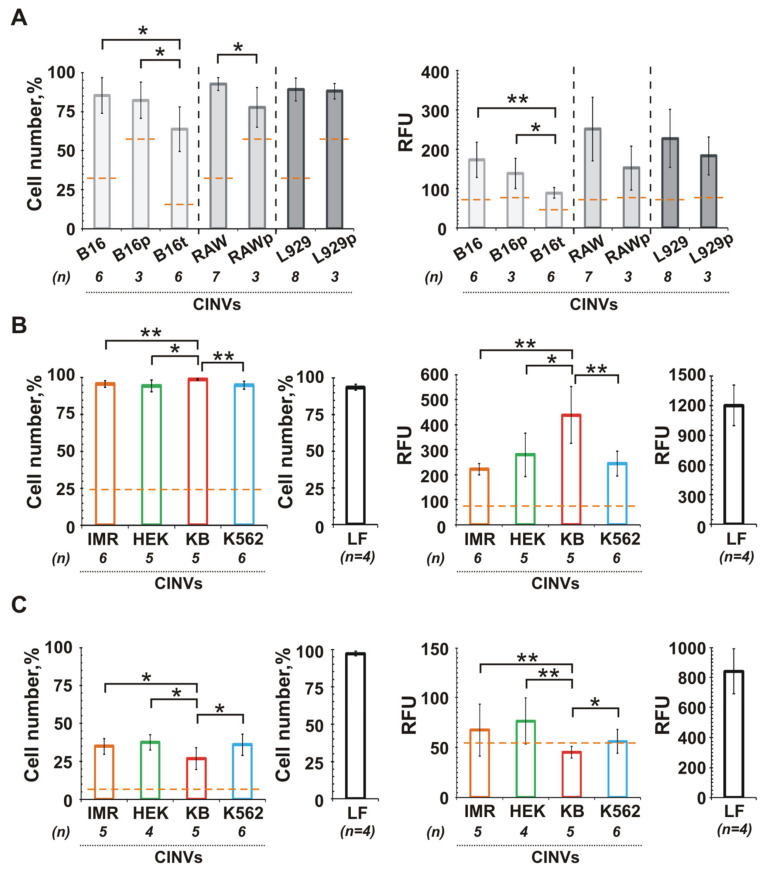
Delivery of FAM-ON mediated by CINVs, derived from various cellular sources. B16 (**A**), KB-3-1 (**B**)**,** and K562 (**C**) cells were incubated with 50 µg FAM-ON loaded CINVs. The origin of CINVs is shown at the bottom of each graph. The effect of surface receptors of recipient cells on CINV uptake was studied for B16 cells (**A**); symbols after the CINV source are as follows: CINVs without prefix (B16, RAW, L929) indicate no cell pre-treatment; CINVs with prefixes “p” and “t” (B16p, RAWp, L929p, and B16t)—cells were pre-incubated with proteinase K or trypsin-EDTA, respectively. The level of FAM-ON self-penetration into the cells (CTRO) is indicated by an orange dashed line. Cell number is the percentage of FAM-positive cells in a population. RFU is relative fluorescence units detected in a cell population. The FAM-ON delivery mediated by Lipofectamine 2000 is indicated as LF. The number of measurements is indicated as *n*. Experimental data are presented as the mean ± SD. Statistical analysis was performed using the Mann–Whitney U test and was carried out between the CINV’s experimental groups (*n* = 3–8); * *p* ≤ 0.05, ** *p* ≤ 0.01.

To reveal factors determining the higher affinity of MSC CINVs to epithelial cancer cells—the source of CINVs or the properties of recipient cells—we investigated the interactions of three types of cancer cells of different histological origins with CINVs derived from various cells (Figure 4).

Melanocytes are cells that originate from the neuronal crest [61,62]. Three cell types were chosen to produce CINVs for interaction with B16 melanoma cells: murine macrophage-like Raw 264.7 cells, mouse L929 fibroblasts, and B16 cells (Figure 4A). Similar levels of FAM-ON accumulation in B16 cells were observed regardless of the CINV origin. Only a slightly higher oligonucleotide accumulation level was observed for RAW CINVs compared to B16 CINVs or L929 CINVs.

Human cervical KB-3-1 carcinoma was analyzed as the second cell type. The entire epithelium of the female reproductive tract, excluding vaginal epithelium, has Müller duct origin, a derivative of the intermediate mesoderm [63]. KB-3-1 cells were incubated with FAM-ON loaded CINVs derived from IMR-32, HEK 293, KB-3-1, and K562 cells (Figure 4B). Similar to the B16 cells, KB-3-1 internalized CINVs of various origins with similar efficiency, although in this case, KB CINVs delivered FAM-ON into KB-3-1 cells more efficiently.

The third type of cancer cells were hematopoietic leukemia K562 cells. These cells most likely originate from hematopoietic stem cells, which originate from the lateral plate mesoderm [64,65]. Similar to KB-3-1, we incubated K562 cells with FAM-ON-loaded CINVs derived from IMR-32, HEK 293, KB-3-1, and K562 cells and observed poor uptake of CINVs by these cells regardless of the CINV source (Figure 4C).

Thus, the data obtained for three cancer cell lines showed that the origin of CINVs was less important for CINV internalization than histological origin and the properties of recipient cells (Figure 4). In order to check whether interactions of CINVs with surface receptors of recipient cells play a role in CINV internalization, we destroyed surface proteins of B16 cells before adding the CINVs, by cell pre-treatment with proteinase K or trypsin-EDTA (Figure 4A). As depicted in Figure 4A, a downward trend in CINV-mediated FAM-ON accumulation was observed in the case of B16 cells pre-incubation with proteinase K (B16p, RAWp, L929p). However, as 37 °C was not the optimal temperature for proteinase K activity, this result was not entirely convincing. We repeated this experiment with B16 cells pre-incubated with trypsin-EDTA and observed an even stronger decrease in the level of CINV internalization, both in terms of the percentage of FAM-positive cells and the mean fluorescence intensity of the cells (Figure 4A; B16t). Thus, these two experiments confirm that interactions with cell surface receptors play an important role in the uptake of CINVs by recipient cells.

### 3.6. Internalization of CINVs Derived from Apoptotic Cells (aCINVs) by Cancer Cells

Investigating the properties of CINVs, we asked whether it is possible to increase the level of CINV internalization by cancer cells without the additional engineering of nanovesicles. Indeed, the level of FAM-ON accumulation mediated by CINVs is not very high and can be increased because the FAM-ON delivery mediated by liposomes provides a significantly higher level of oligonucleotide accumulation in most of the studied cells (Figure 2D and Figure 4).

Here, we examined the cellular uptake of CINVs derived from late apoptotic cells (aCINVs) as a new possibility to improve nanovesicle internalization. We suggested that the transfer of phosphatidylserine to the outer layer of the plasma membrane during apoptosis might have a positive effect on nanovesicle uptake. Moreover, in recent years, there has been interest in the use of EVs isolated from apoptotic cells. For example, DCs stimulated by EVs, derived from dying melanoma cells, have an enhanced anti-cancer effect [66]. Encouraging results were obtained in experiments with several cell cultures, mouse models, and clinical trials following the use of EVs derived from apoptotic cancer cells. These EVs were able to deliver chemotherapeutic agents directly to cancer cells, providing the necessary drug localization in cells, and activating immune cells [67,68,69].

**Figure 5:**
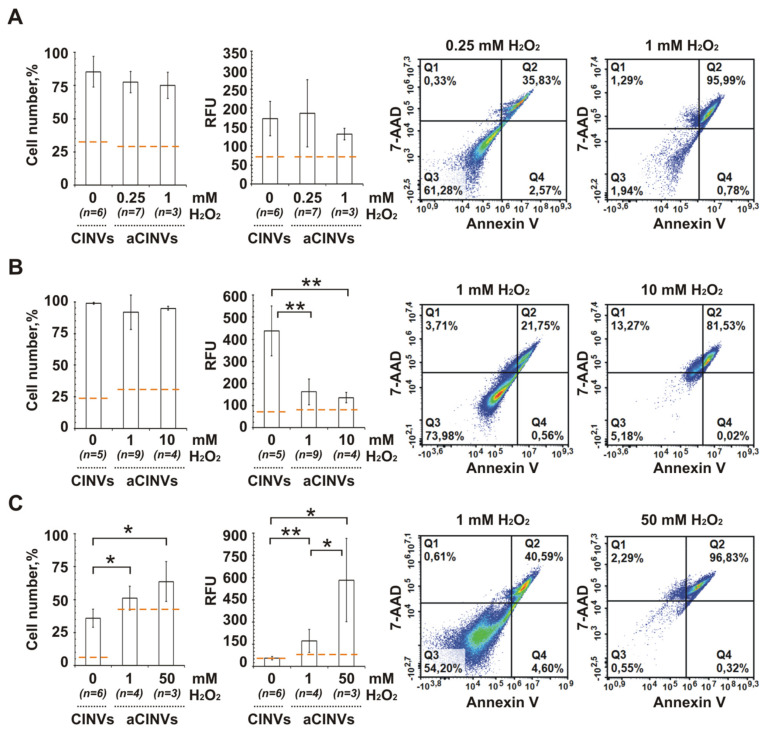
FAM-ON delivery into B16 (**A**), KB-3-1 (**B**), and K562 (**C**) cells mediated by CINVs derived from apoptotic cells (aCINVs). To prepare aCINVs, apoptosis was induced by cell incubation with H_2_O_2_ for 24 h. These aCINVs are indicated on the histograms as used H_2_O_2_ concentration. Data obtained for CINVs, derived from live cells, were used as a control. These CINVs are indicated as 0 mM H_2_O_2_. The histograms show the levels of FAM-ON accumulation in the cells mediated by 50 µg aCINVs or CINVs. B16, KB-3-1, and K562 cells were treated with nanovesicles isolated from the same cell type. Cell number is the percentage of FAM-positive cells in a population. RFU is relative fluorescence units. The level of FAM-ON self-penetration into the cells (CTRO) is indicated by an orange dashed line. Flow cytometry two-parameter plots illustrate the level of cell death induced by H_2_O_2_; cells were co-stained with Annexin V and 7-AAD. Quadrant 1 (Q1) shows necrotic cells. Quadrant 2 (Q2) shows late apoptotic/necrotic cells. Quadrant 3 (Q3) shows live cells. Quadrant 4 (Q4) shows early apoptotic cells. The number of measurements is indicated as *n*. Experimental data are presented as the mean ± SD. Statistical analysis was performed using the Mann–Whitney U test and was carried out between the CINV/aCINV’s experimental groups (*n* = 3–9); * *p* ≤ 0.05, ** *p* ≤ 0.01.

To prepare aCINVs, three types of cancer cells were pre-incubated with various concentrations of hydrogen peroxide for 24 h; based on these experiments, two concentrations of H_2_O_2_ were selected to achieve middle or high levels of apoptotic cells in a population. In Figure 5A–C, flow cytometry data are shown for B16, KB-3-1, and K562 cells to illustrate the number of apoptotic cells in a population after treatment with hydrogen peroxide followed by cell staining with Annexin V/7-AAD: in quadrants 2 (Q2) and 4 (Q4), the number of late apoptotic/necrotic and early apoptotic cells, respectively, is shown.

To induce cell death in B16 cells, they were pre-incubated with either 0.25 mM or 1 mM H_2_O_2_; in KB-3-1 cells–with either 1 mM or 10 mM H_2_O_2_; and in K562 cells–with 1 mM or 50 mM H_2_O_2_. Under these conditions, the number of late apoptotic/necrotic cells (Figure 5) was in the range 36–96% for B16 cells, 22–82% for KB-3-1 cells, and 41–97% for K562 cells. Furthermore, H_2_O_2_ pre-treated cells were used to prepare aCINVs; both aCINV isolation and loading with FAM-ON were performed according to the standard protocol. In addition, it is worth noting that in these experiments, FAM-ON delivery into the cells was mediated by aCINVs isolated from the same cell type. For example, B16 cells were treated with aCINVs isolated from B16 cells.

It was found that aCINV–mediated FAM-ON delivery in B16, KB-3-1, and K562 cells occurred differently than in CINVs. In B16 cells, we observed the tendency to reduce aCINVs uptake compared to CINVs (Figure 5A). In the case of KB-3-1 cells, the pronounced decrease in FAM-ON accumulation was observed when aCINVs were used (Figure 5B). In contrast, K562 cells internalized aCINVs 10-fold more efficiently than CINVs (Figure 5C, 50 mM). In the case of K562 cells, there was the clear advantage of using 50 mM H_2_O_2_-treated cells to produce aCINV compared to using cell pre-treatment with 1 mM H_2_O_2_ (Figure 5C). We hypothesize that the phosphatidylserine-mediated increase in the surface negative charge of these aCINVs was favorable for nanovesicle uptake by K562 cells. Thus, the use of aCINVs might be of interest for delivery of nucleic acid therapeutics to hematopoietic cancer cells; however, further investigations are needed.

### 3.7. Effect of Cyt B in CINVs on Cell Division and FAM-ON Uptake

Finally, we investigated the effect of CINVs on the viability and proliferation of recipient cells. Cyt B used for the preparation of CINVs can be entrapped in lipid structures, such as liposomes [70], or retained in CINVs after their formation. CINVs samples could also contain some residual amount of Cyt B even after the isolation procedure. Because the presence of free Cyt B could be toxic for cells or interfere with the process of CINV internalization by cells, we assessed the Cyt B content in several CINVs samples using mass-spectrometry analysis (MS). We found that the amount of Cyt B in 75 μg (total protein) of CINVs was 2.6 ± 0.6 ng.

Cyt B used for the preparation of CINVs is known to induce apoptosis in various ways. Although the short-term exposure of cells to Cyt B could not be expected to effectively stimulate this process and affect cell viability and CINV internalization, we further analyzed the level of FAM-ON self-penetration into KB-3-1 cells in the presence of 2, 10, or 100 ng Cyt B, corresponding to a similar concentration (2 ng) or significantly exceeding (10 and 100 ng) those achieved during CINV–mediated FAM-ON delivery (Figure S5). Cyt B at these concentrations did not affect the FAM-ON self-penetration into cells. The observed level of FAM-ON accumulation was similar to that of the CTRO, regardless of the Cyt B added. Thus, intracellular accumulation of FAM-ON observed in our studies was mediated by CINVs and was not the artifact arising from sample contamination with Cyt B. These data are consistent with previous findings [15], demonstrating that the short-term (30 min) treatment of cells with Cyt B does not affect cell membrane selective permeability. We observed that the even longer incubation of cells in the presence of Cyt B (4 h in our work) did not affect oligonucleotide self-accumulation in the cells.

**Figure 6:**
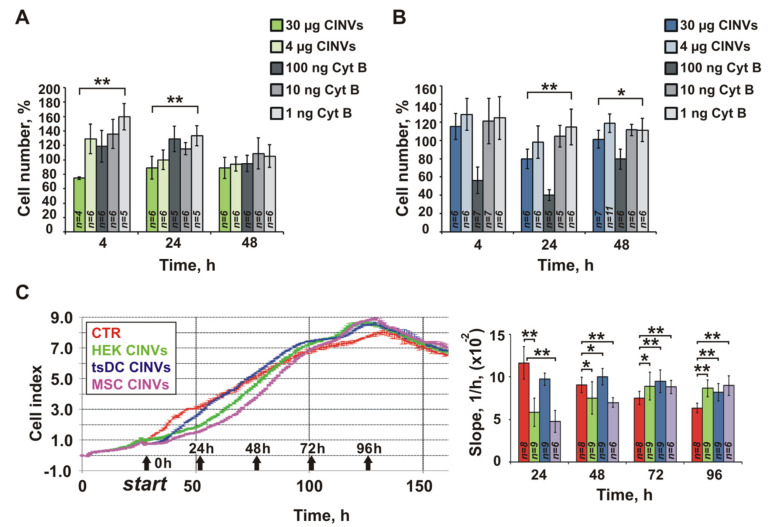
Effect of CINVs on proliferation of A549 (**A**,**C**) and L929 (**B**) cells. (**A**–**B**). WST-1 assay. Cells were incubated with 4 or 30 µg empty CINVs per well or with 1, 10, or 100 ng free Cyt B. Cell proliferation was analyzed after 4 h, 24 h, or 48 h of treatment. The absorbance measured in control (intact) cells was set at 100%. (**C**). Data of real-time impedance-based assay (xCELLigence). Progress curves of A549 proliferation in the presence of 30 µg CINVs of various cellular origins. Cells treated with HEK CINVs—green, tsDC CINVs—blue, MSC CINVs—purple, control cells (CTR)—red. Results were expressed as cell index representative graphs and the rate of cell index change (slope). The number of measurements is indicated as *n*. Data are presented as the mean ± SD. Statistical analysis was performed using the Mann–Whitney U test; * *p* ≤ 0.05, ** *p* ≤ 0.01.

Finally, we analyzed the effect of Cyt B presented in CINV samples on cell viability using the standard WST-1 assay. A549 lung adenocarcinoma cells and L929 fibroblasts were incubated with 4 or 30 µg empty CINVs (without oligonucleotide loading) (Figure 6A,B). The dose of 30 µg CINVs per 150 µL of cell medium was selected as the maximal nanovesicle dose used in FAM-ON delivery experiments (50 µg CINVs per 250 µL), whereas the dose of 4 µg CINVs per 150 µL corresponds to cell-to-CINV ratio in FAM-ON delivery experiments: 50 µg CINVs per 10^5^ cells or 4 µg CINVs per 7500 cells. Cells incubated with free Cyt B were used as a control of Cyt B cytotoxicity: in these experiments, doses of 1, 10, or 100 ng of Cyt B per well (150 µL) were used (Figure 6).

As seen from the data displayed in Figure 6A,B, cell incubation with low doses of free Cyt B stimulate cell division in both studied cell types (1 ng/150 µL and 10 ng/150 µL). This is apparently due to the slight reorganization of the actin cytoskeleton that is favorable for cell division. Indeed, it is known that low concentrations of Cyt B stimulate DNA synthesis [71], whereas high concentrations inhibit DNA replication [21,71]. Dose 100 ng/well of Cyt B was toxic for L929; at this concentration, an almost 2-fold decrease of cell viability was observed (Figure 6B). In contrast, in A549 cells, the effects of 1, 10, or 100 ng Cyt B were similar (Figure 6A). These data additionally confirm that the sensitivity of different cells to Cyt B can vary significantly.

Because 30 µg of CINVs contain approximately 1 ng Cyt B, according to our MS data, one could expect that the effects of 30 µg CINVs and 1 ng of Cyt B on cells would be similar. However, this was not the case (Figure 6A,B); Cyt B at a concentration equivalent to its content in CINVs stimulated cell proliferation compared to control (intact) cells, whereas CINVs during the first 4–24 h slightly decreased the number of viable cells to 75–80% compared to control cells. Thus, we can conclude that the effects of CINVs on the division of recipient cells resulted from other factors, but not from the presence of Cyt B in the sample. Overall, the inhibitory effect of CINVs on cell proliferation was observed during 4–24 h and expired after longer observations. It is also worth noting that this inhibitory effect was stronger in the case of A549 cells than for L929 cells.

To follow the effects of CINVs on cell division more precisely, we studied the proliferation of A549 cells in the presence of 30 µg CINVs derived from various cellular sources by real-time impedance-based assay (xCELLigence) (Figure 6C) and observed a decrease in the rate of cell division (slope) during the first 24–48 h compared to control cells (CTR). However, the retardation of division in CINV-treated cells was also temporal and the rate of A549 proliferation was restored by 72 h, reaching the level of control cells (Figure 6C). Thus, the effect of CINVs on cell proliferation was reversible and only a temporal delay in cell division was observed.

## 4. Discussion

### 4.1. Isolation and Characterization of EVs and CINVs

In this work, we isolated and compared physical parameters of EVs and CINVs obtained from various types of cells. The size of nanocarrier is one of the most important parameters for the efficient delivery of therapeutics into solid tumors [72,73,74]. Their diameter should be greater than 10 and less than 200 nm. This allows nanovesicles to avoid fast elimination by the kidney and reduces the activation of the complement system and nanovesicle accumulation in the spleen and liver [72,73,74]. We analyzed the sizes of the obtained nanovesicles by DLS, NTA, and TEM and observed good agreement between TEM and NTA data in contrast to DLS, where nanovesicle sizes were overestimated. As DLS tends to better evaluate the size of larger particles, suppressing the smaller particles in a size distribution, this may be the cause of observed differences. We concluded that the diameters of both EVs and CINVs mainly did not exceed 150 nm, which was required for their application as a drug delivery system.

Other important parameters of the nanocarrier are the simplicity of preparation and yield. Among the different methods for nanovesicle quantifying [75], the measurements of the total protein concentration and NTA are commonly used. Comparison of the yields of EVs and CINVs can be performed correctly only for one cell type, as the yield of EVs strongly depends on the type of secreting cell. In contrast, the yield of CINVs was similar for different cell types (see Section 2.5, Materials and Methods). It should be noted that the yield of CINVs derived from apoptotic cells (aCINVs) was 1.5- to 4-fold lower compared to live cells (total protein), which was likely associated with a decrease in cell volume during apoptosis progression.

The use of total protein measurement alone to compare the yields of EVs and CINVs can lead to a false conclusion. Therefore, in this study, one milligram (total protein) of tsDEX was produced from 17.7 ± 6.5 × 10^8^ cells, which was 12-fold higher than needed to obtain the same amount of tsDC CINVs (1.5 ± 1.1 × 10^8^ cells/mg). However, according to NTA data (Table 1), 10^12^ tsDEX were produced from approximately 10^9^ cells and 10^12^ tsDC CINVs from 3–7.5 × 10^8^ cells. Thus, the yields of these nanovesicles were similar.

Maintaining the integrity of the nanocarrier structure is one of the necessary quality criteria of drug delivery vectors. Here, we observed that the surface proteins of tsDC-derived nanovesicles (tsDEX or tsDC CINVs) were partially damaged. Apparently, the manner of nanovesicle isolation was not responsible for this, as surface determinants on both tsDEX and tsDC CINVs were damaged. In contrast, the cells used for the production of both EVs and CINVs could influence the nanovesicle characteristics. Indeed, the MSC-derived CINVs retained their surface protein architecture well, which suggested their suitability for the creation of CINVs. These data are in good agreement with previous observations [36,46,76].

### 4.2. Cytotoxicity of CINVs

To assess the biological safety of CINVs, we first analyzed the presence of Cyt B in CINVs after preparation. The level of Cyt B was found to be 2.6 ± 0.6 ng per 75 µg CINVs (total protein). We observed that CINV themselves, but not equal amounts of free Cyt B added to the cell medium, caused 24 h retardation of cell division. Cell proliferation was decreased by 20–25% during the first 24 h after treatment with 30 µg CINVs and then recovered to the level of control (Figure 6A,B). Similarly, the CINV-treated cells restored the rate of the division after 24 h of retardation; by 72 h, cells treated with CINVs had a rate of division similar to that of the control at 48 h (Figure 6C).

Different mechanisms could underline the effect of CINVs on cell proliferation. First, it is possible that the intrinsic content of the CINVs may affect cell proliferation. Indeed, we observed some influence of CINV origin on the rate of cell division (Figure 6C), but the tendency towards delay was observed in all cases (Figure 6C). Second, it is possible that nanovesicles obtained from cells with disorganized actin filaments are characterized by less rigidity, which may be the main reason of their effective internalization by cancer cells [77]. Fusion of CINV membrane with cells may lead to temporary destabilization of cell plasma membrane resulting in retardation of cell proliferation.

### 4.3. Interaction of DC-Derived EVs and CINVs with Cells

The use of cell membrane-derived artificial nanovesicles shows that their biological effects can be similar to EVs or different from them [46,78,79,80]. Differences in the action of natural and artificial nanovesicles arise from the differences in their protein profiles [81]. In this work, we investigated the interaction of DC-derived EVs and CINVs with various types of cells and observed that their tropism coincided only partially. The EVs isolated from the conditioned medium of tsDCs (tsDEX) were only internalized by some of the tested cells, whereas tsDC CINVs were taken up by all of the studied cells with similar efficiency. Considering that the surface proteins of tsDC CINVs were partially damaged, it is quite possible that their low selectivity was due to the disturbed interaction between nanovesicles and recipient cells.

### 4.4. Endometrial MSCs as Sources of CINVs

MSCs are highly demanded cells for the production of both natural EVs and their mimetics because these cells are able to home on to damaged tissue or to a tumor microenvironment [82,83,84], and the MSC-derived nanovesicles can possibly inherit these properties. A recent study showed that MSC-derived EVs have advantages in cancer therapy compared to tumor-derived vesicles [85]. However, the dual role of MSCs in tumor progression still limits their practical use [82,83,84]. Indeed, both mimetic vesicles and EVs obtained from MSCs will transfer the intrinsic contents of these cells and thereby influence the fate of tumor cells. Today, researchers have concluded that the tissue source of MSCs may be the key to unraveling their antagonistic effects [83]. Based on this, the choice of MSC origin is the primary task for the creation of nanovesicles.

Here, we used human MSCs, obtained from the functional layer of endometrium, to produce CINVs and treat different cancer cells. Endometrial MSCs are characterized by rapid division and capacity to multilinear differentiation. These cells and MSCs obtained from menstrual blood are attractive for regenerative medicine [86]. Unfortunately, the role of endometrial MSC in cancer development has not been studied. The MSC CINVs used in this work interacted with epithelial cancer cells more effectively. Although we suggest that the origin of CINVs only slightly influences the preference of cells to interact with them, it cannot be completely ruled out that the endometrial MSCs may be favorable for targeting to epithelial cancers. At the same time, this bold conclusion must be verified in the future.

### 4.5. CINVs in Cancer Treatment

The choice of a cellular source to produce EVs or their mimetics is usually considered an opportunity to target nanovesicles to cells of interest. For example, macrophages have recently been used as a source of nanovesicles for binding to cancer cells [87,88]. At the same time, recipient cells are not passive participants in this process and, together with the microenvironment, can dictate the manner of internalization of nanovesicles [7,8].

In this work, we investigated the influence of CINV origin on the efficiency of their internalization by recipient cells. We incubated three cancer cells (melanoma, cervical cancer, and leukemia cells) with CINVs derived from different cell sources. We found that the level of CINV uptake in cancer cells was similar regardless of the origin of the nanovesicles. In the case of melanoma cells, this interaction was dependent on the integrity of the cell surface proteins. Thus, we assume that the properties of the recipient cells play a more important role in the uptake of CINVs compared to the source of CINVs.

We also investigated the uptake of aCINVs isolated from the late apoptotic cells. During apoptosis, anionic phosphatidylserine is externalized to the outer layer of the plasma membrane [89], which causes an increase in the surface negative charge of such nanovesicles. We hypothesize that the differences observed in aCINV internalization by melanoma cells, cervical cancer, and myelogenous leukemia cells demonstrated their capacity to interact and internalize nanovesicles with increased surface negative charge. We found a significant increase in aCINV internalization by K562 leukemia cells and a strong negative effect on the uptake of aCINVs by cervical cancer cells. We assume that aCINVs may be of interest for the treatment of blood cancer cells, which will require further research. Moreover, there is a growing interest in the development of a drug delivery system based on the apoptotic mimetic vesicles targeted to tumor-associated macrophages [90]. We suggest that the in vivo study of aCINVs may be of interest for this purpose.

### 4.6. Limitaions of This Research

The current study indicates the high level of contamination of EV samples with non-vesicular protein in both tsDEX and imDEX preparations. Given that, in the future the protocol of EV isolation and purification could be optimized. In addition, the EV staining with negative biomarkers [91] should be performed in the case of EV based drug delivery in vivo.

We observed that both EVs and CINVs were highly heterogeneous (PdI, Table 1), and CINVs tended to form a large number of aggregates under conditions used. Although the use of other storage conditions (serum, as an example [38,39]) can stabilize CINVs, this can be a factor of additional contamination of CINV samples and seems to be undesirable for their further clinical use. In addition, standardization of CINV size is likely to require modification of their preparation technology.

Cell-derived biomimetic vesicles are usually obtained by mechanical or chemical destruction of cells. They are not naturally secreted by cells, as with EVs. Despite the growing interest in this type of therapeutic carrier, there are no quality standards to assess their integrity and purity. In the current study, the minimal parameter for CINV assessment was their size; however, we expect the criteria for CINV characterization will be expanded and formulated in the near future.

A further study of CINVs will be focused on their in vivo application. This requires consideration of many parameters, including the source of nanovesicles, the route of their administration, the choice of an animal model, and the way of nanovesicles labelling, as all of them can affect nanovesicle biodistribution [92]. To date, the problem of nonspecific accumulation of the lipid-based nanocarrier in the liver, lungs, and other organs has not been solved. In addition, to decrease the phagocytic clearance, the CINVs must also be modified, which can increase their immunogenicity [93]. Whether the cellular tropism of CINVs observed in this study will be preserved in animal experiments will be elucidated in the future.

## 5. Conclusions

Cancer is one of the most widespread and difficult to treat human diseases, requiring early diagnosis and long-term therapy. Today, nanoparticles of various natures are being developed to encapsulate or bind chemotherapeutic or other drugs and deliver them to certain types of cancer cells with minimal negative side effects. Unfortunately, many highly efficient delivery systems still suffer from disadvantages, such as the development of chronic or acute inflammation, instability or rapid elimination from the blood, high toxicity to the liver and kidneys, and the inability to overcome complex biological barriers. In addition, the high rate of adaptation of cancer cells to external influences requires the improvement of existing therapeutic solutions.

The EV-based drug delivery system is of great interest due to the possible application of EV’s natural ways of internalization for targeted delivery. In fact, the isolated EVs also require additional modifications for internalization in the tissues of interest. It is quite possible that this need is due to the limitations of the methods used for EV isolation, which may change in the future. Given the complex procedure of EV preparation and purification, attempts are being made to create vesicle mimetics with similar biological properties and a standardized preparation procedure. Liposomes, particles covered with cell membranes, nanovesicles obtained by cell extrusion, hybrids of liposomes and EVs, and CINVs are not yet a complete arsenal of lipid-based delivery systems that are being adapted to cancer therapy.

In this study, we analyzed the tropism of cytochalasin-B-inducible nanovesicles (CINVs) of various cellular origins in different cells in vitro. We found that CINV internalization mainly depends on the feature of recipient cells, including retention of the integrity of cell surface receptors. The aCINVs, derived from late apoptotic/necrotic cells, demonstrated different CINV patterns of internalization by cells, which was probably due to their higher surface negative charge.

The potential of CINVs for the delivery of therapeutic compounds has been demonstrated for chemotherapeutic, photosensitizing, and nucleic acid drugs. We have previously found that CINVs could be loaded with short DNA oligonucleotides and used for nucleic acid delivery into recipient cells. The efficiency of nanovesicle internalization was high enough and we continued to study their biological properties. The next step in the study of CINV application for nucleic acid delivery should be devoted to investigation of the biological effect of these preparations. In addition to short DNA oligonucleotides, CINVs can be examined for delivery of other types of nucleic acids, such as siRNA or large sequences, including mRNA or CRISPR, which are difficult to load.

## Figures and Tables

**Table 1:** Characteristics of EVs and CINVs used in this study.

Vesicles	Cells	Abbr.	Protein Markers	Diameter [Ø], nm				Particles (×10^12^) per mg of Total Protein
				DLS		NTA, Mean Ø	TEM Ø ≤ 150	
				PdI	Z-Average			
**EVs**	Bone marrow DCs	imDEX	CD63^+^; CD81^+^; CD86^+^; CD80^−^; MHC II^+^	0.27	199 ± 6	100–119	91%	0.8–3.7
	tsDCs	tsDEX	CD63^+^; CD81^+^; CD86^+^; CD80^−^; MHC II^−^	0.38	203 ± 4	95–117	87%	1.8
**CINVs**	tsDCs	tsDC	CD63^+^; CD81^−^; CD86^+^; CD80^−^; MHC II^−^	0.47	390 ± 21	113–129	90%	0.2–0.5
	MSCs	MSC	CD14^−^; CD34^−^; CD45^−^; CD19^−^; CD105^−^; CD73^+^; CD90^+^; CD9^+^; CD63^+^; CD81^+^	0.46	304 ± 23	92–117	82% [26]	0.4–1.0
	HEK 293	HEK	–	0.59	483 ± 76	125–144	66%	0.2–0.3
	IMR-32	IMR	–	0.69	599 ± 124	130–144	66%	0.3
	KB-3-1	KB	–	0.53	396 ± 26	–	–	–
	RAW 264.7	RAW	–	0.69	886 ± 270	–	–	–
	L929	L929	–	0.55	442 ± 113	–	–	–
	K562	K562	–	0.60	648 ± 88	–	–	–
	B16	B16	–	0.55	593 ± 113	–	–	–

Abbr–abbreviation. DLS data [Ø] are presented as mean ± standard deviation (SD). PdI–polydispersity index. The mg of nanovesicles was measured as total protein concentration. The number of particles in samples was measured by NTA in 1 mg (total protein) of EVs or CINVs diluted in 1 mL of buffer. “-“–not determined.

## Data Availability

Not applicable.

## Supplementary Materials

The following are available online at https://www.mdpi.com/article/10.3390/pharmaceutics13111911/s1, Figure S1: Phenotypes of DC-derived EVs (A), tsDCs (B) and CINVs (C); Figure S2: Phenotype of MSC CINVs; Figure S3: Accumulation of fluorescein-labelled single-stranded DNA-oligonucleotide (FAM-ON) in HEK 293 cells mediated by MSC CINVs; Figure S4: Confocal microscopy analysis of FAM-ON delivery into HEK 293 cells mediated by MSC CINVs; Figure S5: FAM-ON uptake by KB-3-1 cells in the presence of various amounts of Cyt B; Table S1: Treatment of CINVs loaded with FAM-ON with mung bean nuclease.

## Abbreviations

Ø–diameter; aCINVs–CINVs derived from apoptotic cells; CI_95_–95% confidence interval; CINVs–cytochalasin-B-inducible nanovesicles; CTR–control; CTRO–control of FAM-ON self-penetration into cells; Cyt B–Cytochalasin B; DCs–dendritic cells; DLS–dynamic light scattering analysis; EVs–extracellular vesicles; FAM-ON–fluorescein-labelled single-stranded DNA-oligonucleotide; imDEX–EVs derived from immature bone marrow DCs; LF–FAM-ON delivery by Lipofectamine 2000; MS–mass spectrometry analysis; MSCs–mesenchymal stem cells; n–a number of measurements; NTA–nanoparticle tracking analysis; PdI–polydispersity index; RFU –relative fluorescence units; SD–standard deviation; TEM–transmission electron microscopy; tsDEX–EVs derived from tsDCs.
