# Supplementary material for: Tropism of Extracellular Vesicles and Cell-Derived Nanovesicles to Normal and Cancer Cells: New Perspectives in Tumor-Targeted Nucleic Acid Delivery
Source: Pharmaceutics. 2021 Nov 11;13(11):1911. doi: 10.3390/pharmaceutics13111911 (PMC8621453; doi:10.3390/pharmaceutics13111911)
Supplement: Supplementary file 1 [file pharmaceutics-13-01911-s001.zip › pharmaceutics-1394834-supplementary.pdf]

pharmaceutics

MDPI

Supplementary Materials: Tropism of Extracellular Vesicles and Cell-Derived Nanovesicles to Normal and Cancer Cells: New Perspectives in Tumor-Targeted Nucleic Acid Delivery

Anastasiya Oshchepkova, Oleg Markov, Evgeniy Evtushenko, Alexander Chernonosov, Elena Kiseleva, Ksenia Morozova, Vera Matveeva, Lyudmila Artemyeva, Valentin Vlassov and Marina Zenkova *

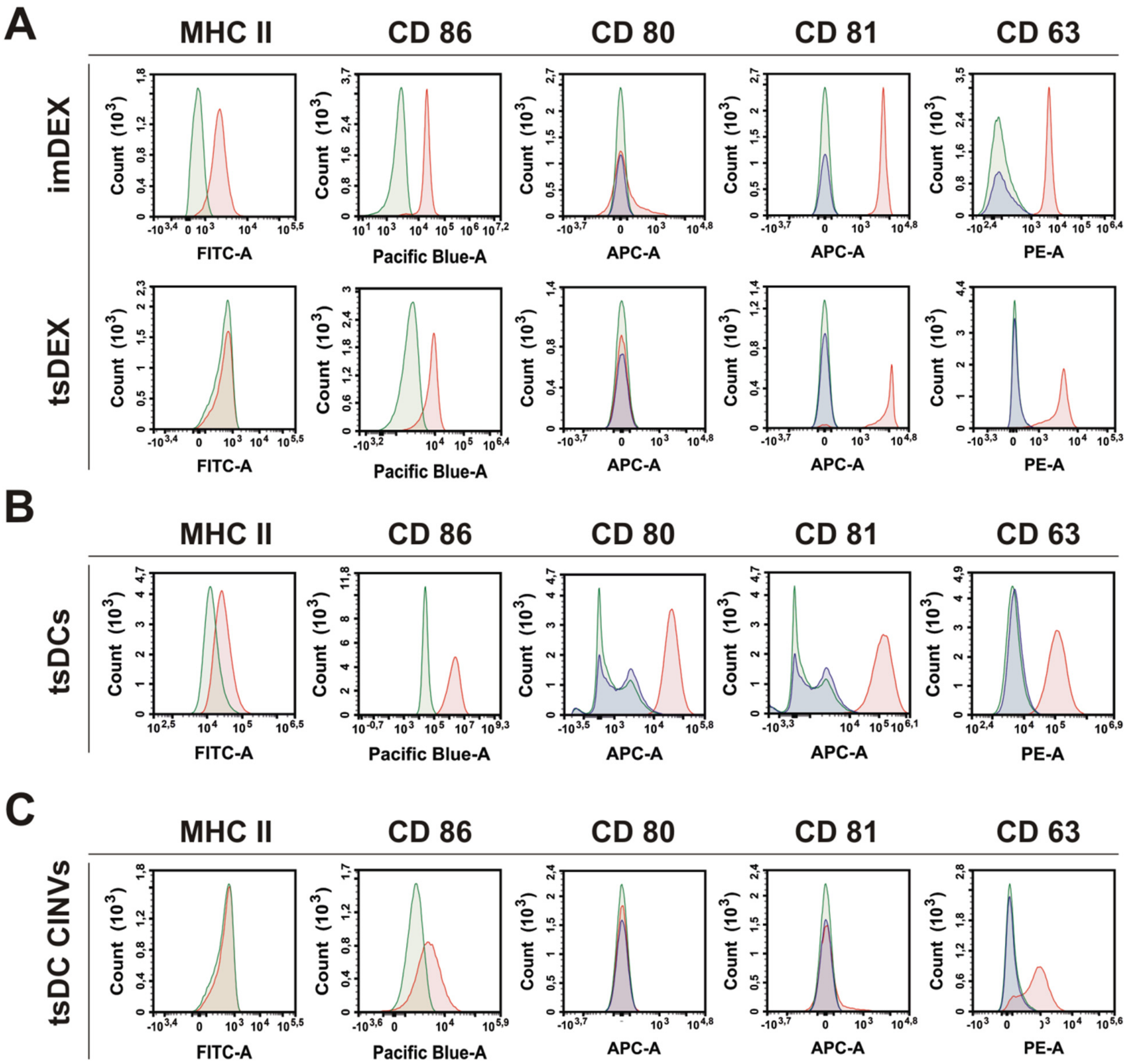

Figure S1. Phenotypes of DC-derived EVs (A), tsDCs (B) and CINVs (C). The EVs derived from bone marrow-derived DCs are indicated as imDEX; the EVs derived from tsDCs are indicated as tsDEX; the CINVs derived from tsDCs are indicated as tsDC CINVs. To analyze the surface proteins of EVs or CINVs, nanovesicles were immobilized on aldehyde/sulfate latex beads and stained with specific antibodies or isotype controls. Green color indicates control unstained latex beads or cells, blue color – staining with corresponding isotype control and red color – staining with protein-specific antibodies.

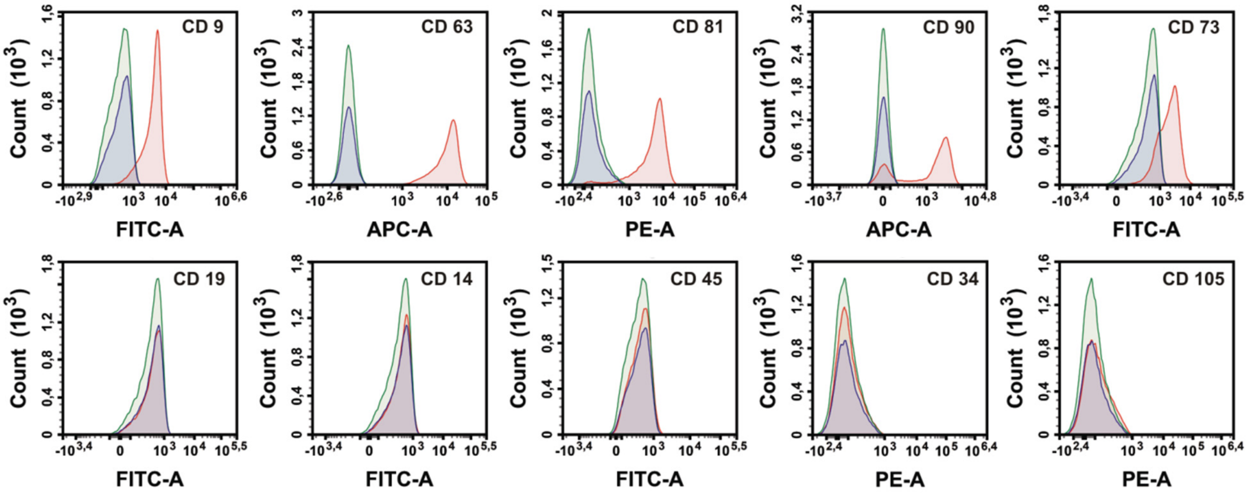

Figure S2. Phenotype of MSC CINVs. CINVs were immobilized on aldehyde/sulfate latex beads and stained with specific antibodies or isotype control. Green color indicates unstained latex beads, blue color – staining with corresponding isotype control and red color – staining with protein-specific antibodies.

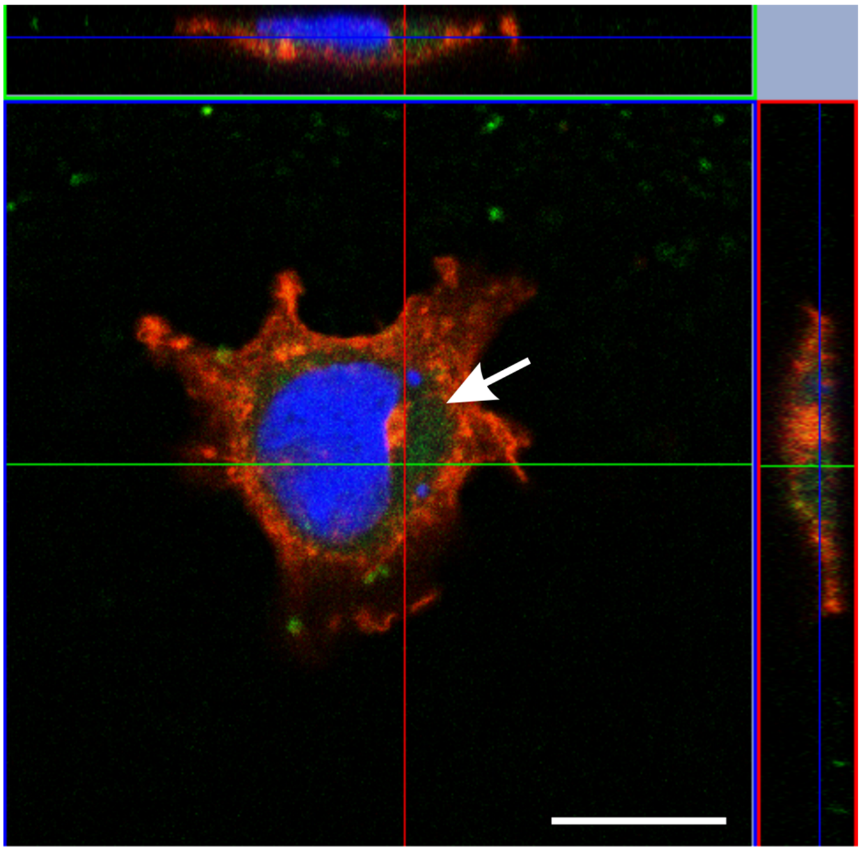

Figure S3. Accumulation of fluorescein-labelled single-stranded DNA-oligonucleotide (FAM-ON) in HEK 293 cells mediated by MSC CINVs. Confocal microscopy, Z-stack image. Intracellular accumulation of FAM-ON is indicated by white arrow. Nuclei are indicated by blue color, actin filaments – by red color. The CINVs loaded with FAM-ON were indicated as green signal. Scale bar = 20 μm.

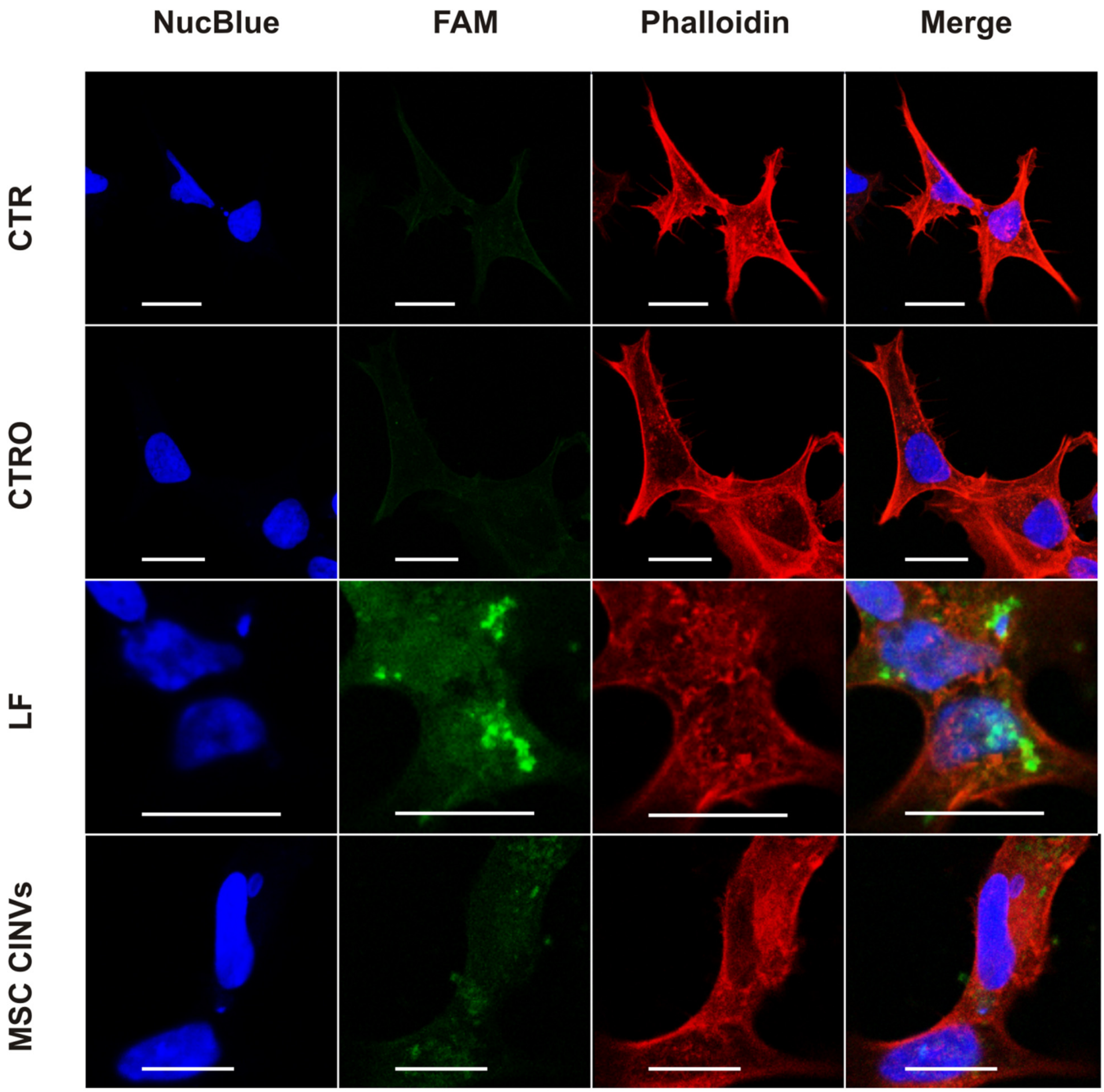

Figure S4. Confocal microscopy analysis of FAM-ON delivery into HEK 293 cells mediated by MSC CINVs. Control untreated cells are indicated as CTR. The level of FAM-ON self-penetration is indicated as CTRO. The FAM-ON delivery mediated by Lipofectamine 2000 is indicated as LF. All images in one channel were made in identical settings. Scale bar = 20 μm.

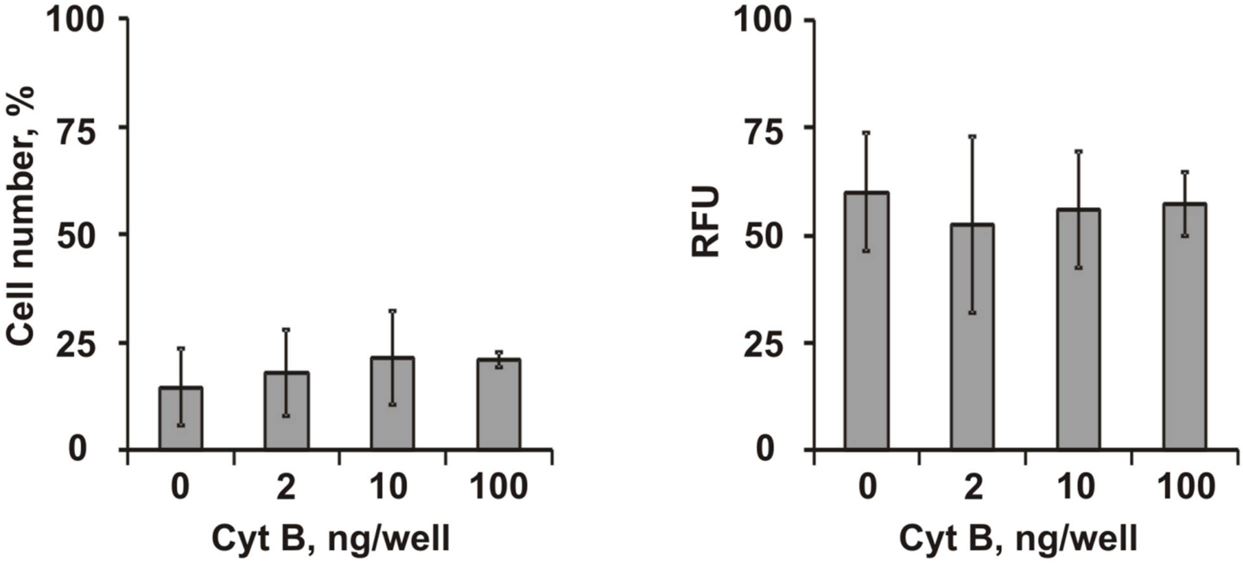

Figure S5. FAM-ON uptake by KB-3-1 cells in the presence of various amounts of Cyt B. KB-3-1 cells (10^5 cells/well) were incubated with 1 nmol FAM-ON in the absence (0 ng) or presence of (2, 10 or 100 ng) Cyt B in DMEM supplemented with 10% EV-depleted FBS (V = 250 μl) for 4 h. Data are presented as the mean ± SD. Cell number is the percentage of FAM-positive cells in a population. RFU – relative fluorescence units detected in a cell population. Experiments with 2 ng, 10 ng or 100 ng Cyt B were twice independently repeated. Experiments without Cyt B (0 ng) were carried out 6 times.

Table S1. Treatment of CINVs loaded with FAM-ON with mung bean nuclease.

CINVs	CTR			CINVs	Mung bean nuclease		
	%	RFU	n		%	RFU	n
**MSC**	95.1 ± 0.6	39.8 ± 2.7	3	**MSC**	95.5 ± 1.6	34.5 ± 5.4	3
**HEK**	93.7 ± 1.3	10.5 ± 0.9	3	**HEK**	94.3 ± 1.5	10.4 ± 1.5	3

To confirm the internal localization of FAM-ON, loaded into CINVs, the samples were treated with mung bean nuclease (see Materials and Methods). In parallel control untreated samples (CTR) were analyzed. To detect the level of FAM-signal in CINVs, nanovesicles were immobilized on aldehyde/sulfate latex beads and analyzed by flow cytometry. Data are presented as the mean ± SD. The percent (%) indicates FAM-positive latex beads, containing CINVs loaded with FAM-ON. RFU – relative fluorescence units. In each condition three samples (n) were analyzed.
